# Silent Partners in the Mill: Unveiling the Role of Additives in Mechanochemical Synthesis

**DOI:** 10.1002/chem.202503536

**Published:** 2026-01-05

**Authors:** Johanna Breinsperger, Nika Podlesnik, Francesco Mele, Michael Schnürch

**Affiliations:** ^1^ Institute of Applied Synthetic Chemistry TU, Vienna Wien Austria; ^2^ SynCat Lab, Department of Chemistry, Life Sciences and Environmental Sustainability University of Parma Parma Italy

**Keywords:** additive, energy transfer, mechanochemistry, salt effect, solvent free

## Abstract

Mechanochemistry has attracted significant attention as a sustainable and efficient alternative to solution‐based synthesis, offering the advantage of solvent‐free conditions or the use of only minor amounts of solvent. Many established mechanochemical transformations rely on the use of additives—a strategy broadly referred to as additive‐assisted grinding. The most employed additives are liquids (liquid‐assisted grinding (LAG)), ionic solids, and non‐ionic additives. Additionally, ionic liquids (ionic liquid‐assisted grinding (IL‐AG)), piezoelectric, or mechanoluminescent materials can be used. This review provides an overview of additive‐assisted organic synthetic transformations under mechanochemical conditions, highlighting the roles, advantages, and limitations of additives, as well as emerging trends from recent literature.

## Graphical abstract

Mechanochemistry offers a sustainable, solvent‐free alternative to traditional synthesis, often enhanced by additives in a process known as additive‐assisted grinding. This review discusses how various additives, including liquids, ionic solids, and advanced materials like ionic liquids and piezoelectric compounds, improve reaction efficiency, highlighting their benefits, limitations, and recent developments.

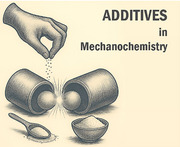

## 1 Introduction

Mechanochemistry has emerged as a powerful and sustainable alternative to traditional solution‐based synthesis, enabling chemical transformations through the direct application of mechanical energy. By circumventing or minimizing the need for bulk solvents, mechanochemical methods drastically reduce waste and improve reaction efficiency, aligning closely with the principles of green chemistry [1]. Over the past decade, the field has expanded rapidly, driven by the development of sophisticated milling technologies [2, 3] and a growing understanding of how reaction conditions influence outcomes. Mechanochemistry has progressed from simple manual grinding with a mortar and pestle to a range of automated technologies capable of delivering controlled and reproducible mechanical energy. Ball milling represents the most widely used approach in mechanochemistry: reagents and grinding media are sealed in a jar, and reactions proceed through repeated impacts or shear forces, with vibratory mills promoting high‐frequency collisions and planetary mills generating more intense shear through combined rotational motions [1]. In recent years, technologies such as twin‐screw extrusion (TSE) [4, 5] and resonant acoustic mixing (RAM) [3, 6] have also been explored, further expanding the methodological landscape, from scalable continuous processing to media‐free protocols. Together, these tools offer a versatile platform for mechanochemical synthesis. Central to this progress has been the recognition of additives—liquids, ionic materials, and non‐ionic solids—as crucial, though still mechanistically obscure, components that modulate reactivity, selectivity, and scalability. These “silent partners” not only facilitate energy transfer and mixing but can also act as catalysts, stabilizers, or selectivity‐directing agents. As a result, additive‐assisted mechanochemistry is redefining synthetic possibilities, bridging the gap between efficiency, tunability, and environmental responsibility [7, 8]. Owing to these features, mechanochemistry has become a powerful tool for both laboratory‐scale synthesis [9] and industrial applications [10], offering significant advantages in terms of atom economy, energy efficiency, and safety [7].

Additives in mechanochemical reactions can serve multiple functions, ranging from active roles, such as base, acid, catalyst, or redox partner, to more passive but essential purposes, such as simply acting as grinding/bulking agents or dilutants. The purpose of additives being used as grinding auxiliaries is to improve mixing/material transfer and aiding energy transfer, especially if the reagents used are liquids.

This review provides an overview of the various types of additives, classified according to their chemical nature. Further, representative examples on recent developments are included, where these additives play a crucial role in the reaction outcome or in some cases serving as only bulking material. With this work, we aim to provide insights that can help guide the choice of additive in specific mechanochemical reactions aiding in the broader development of mechanochemistry as a sustainable and predictable synthetic methodology.

**FIGURE 1:**
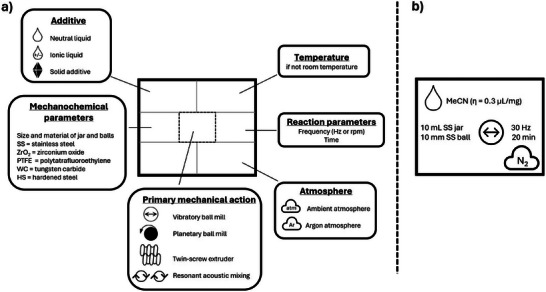
Schematic representation of a mechanochemical reaction.

To increase consistency and encourage the utilization of a uniform schematic representation for mechanochemical reactions, this review employs the format proposed by Michalchuk and Boldyreva [11] (Figure 1a). It includes a box below the reaction arrow in which parameters such as the primary mechanochemical action, material of both jar and balls, milling time and frequency, atmosphere and additives are reported. As an example, Figure 1b describes a reaction conducted in a 10 mL stainless steel (SS) jar, containing a 10‐mm‐diameter ball of the same material, subjected to milling in a vibratory ball‐mill at a frequency of 30 Hz for 20 min under nitrogen atmosphere, and acetonitrile (MeCN) as a liquid additive.

### 1.1 Liquid‐Assisted Grinding

Although mechanochemistry aims to eliminate solvents, the deliberate addition of small amounts of liquid is frequently employed to improve reactivity, accelerate transformations, and tailor product properties. This strategy, commonly referred to as liquid‐assisted grinding (LAG), involves introducing a certain quantity of liquid during mechanochemical processes to influence the course of the reaction [12, 13]. The extent of liquid addition is typically expressed by the η parameter (µL/mg), defined as the ratio of liquid volume to the total mass of reagents. Depending on the system, LAG can act in a catalytic fashion [14], increase product crystallinity [15, 16, 17, 18], or polymorph formation [19, 20, 21, 22]. Rarely, the liquid acts only as a mixing aid, and shows little influence on its nature [23]. Typically, the choice and amount of liquid added directly dictates the reaction efficiency and outcome [2, 24, 25, 26, 27, 28, 29, 30, 31, 32, 33, 34, 35], as in certain cases, even changes in product selectivity have been observed [2, 36, 37, 38], underscoring the significance of the liquid additive as a reaction variable. As this subject is extensive and has been reviewed in detail elsewhere, we limit ourselves to this brief overview and refer the reader to dedicated reviews [12, 13, 39, 40, 41, 42] for a more comprehensive discussion.

### 1.2 Ionic Liquid‐Assisted Grinding

Ionic liquids (ILs) attracted interest due to their polarity, negligible vapor pressure, low flammability, high thermal stability, wide electrochemical windows, and high structural tunability. Their low miscibility with many organic solvents facilitates recovery and recycling, making them appealing as “green” alternatives to conventional solvents [43, 44, 45]. Traditionally, ILs are synthesized in solution [46] or under heating [47]. Recent advances in mechanochemistry have enabled alternative solvent‐free routes [48]. However, their application in mechanochemical organic synthesis remains limited, with most studies employing ILs as additives in co‐crystal [49] formation and in the synthesis of metal organic frameworks (MOFs) [50].

**FIGURE 2:**
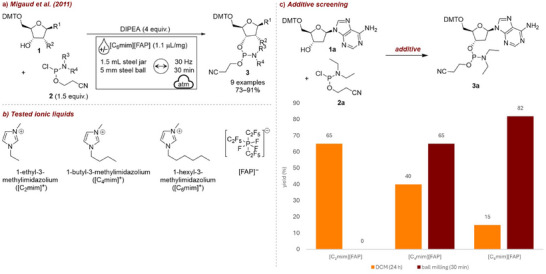
(a) Mechanochemical phosphitylation of nucleosides with chlorophosphoramidites employing ILs. (b) Structures of tested IL additives. (c) Additive screening. Mechanochemical reaction conditions: **1a** (50–60 mg), **2a** (1.5 equiv.), DIPEA (4 equiv.), *additive* (2.25 equiv.), 1.5 mL steel jar, 5 mm steel ball, mixer mill: 30 Hz, 30 min. Reaction conditions in solution: **1a** (50–60 mg), **2a** (2.5 equiv.), DIPEA (4 equiv.), [C_n_mim][FAP] (2.5 equiv.), in dry DCM, 24 h.

In 2011, Migaud et al. [51] reported the first integration of ILs as liquid additives under mechanochemical conditions. They achieved the phosphitylation of nucleosides **1** (Figure 2a) with various chlorophosphoramidites **2**. In this context, mechanochemistry enabled reactions with poorly soluble species, while ILs acted as protective media for hydrolytically sensitive intermediates [52]. Notably, the group obtained excellent yields even with small dialkylamino and diisopropylamino groups, which had been a major limitation in earlier studies. To probe the influence of IL‐substrate interactions, the reactions were carried out with tris(pentafluoroethyl)trifluorophosphate ([FAP]^–^)‐based ILs with varying imidazolium alkyl chain lengths (ethyl, butyl and hexyl, Figure 2b) under ball milling for 30 min or stirring in dichloromethane (DCM) for 24 h (Figure 2c). The authors observed that in the presence of shorter alkyl chain ILs the yield of the product was higher when the reaction was conducted in solution. In contrast, when the reactions were carried out in the presence of longer chain ILs [C_4_mim][FAP] and [C_6_mim][FAP], ball milling proved to be the superior technique. After only 30 min of milling, isolated yields of 65% to 82% were obtained for the model reaction, compared with <40% after 24 h under standard stirring. Under mechanochemical conditions, these reactions consistently afforded high isolated yields (73%–91%) for products previously inaccessible by conventional stirring in DCM. The hydrolytically unstable derivatives could be stored in the presence of small amounts of IL at room temperature under air, remaining stable for at least 5 days. This work highlights the dual role of ILs as both stabilizing additives during mechanochemical synthesis and protective media for storage of sensitive products.

**FIGURE 3:**
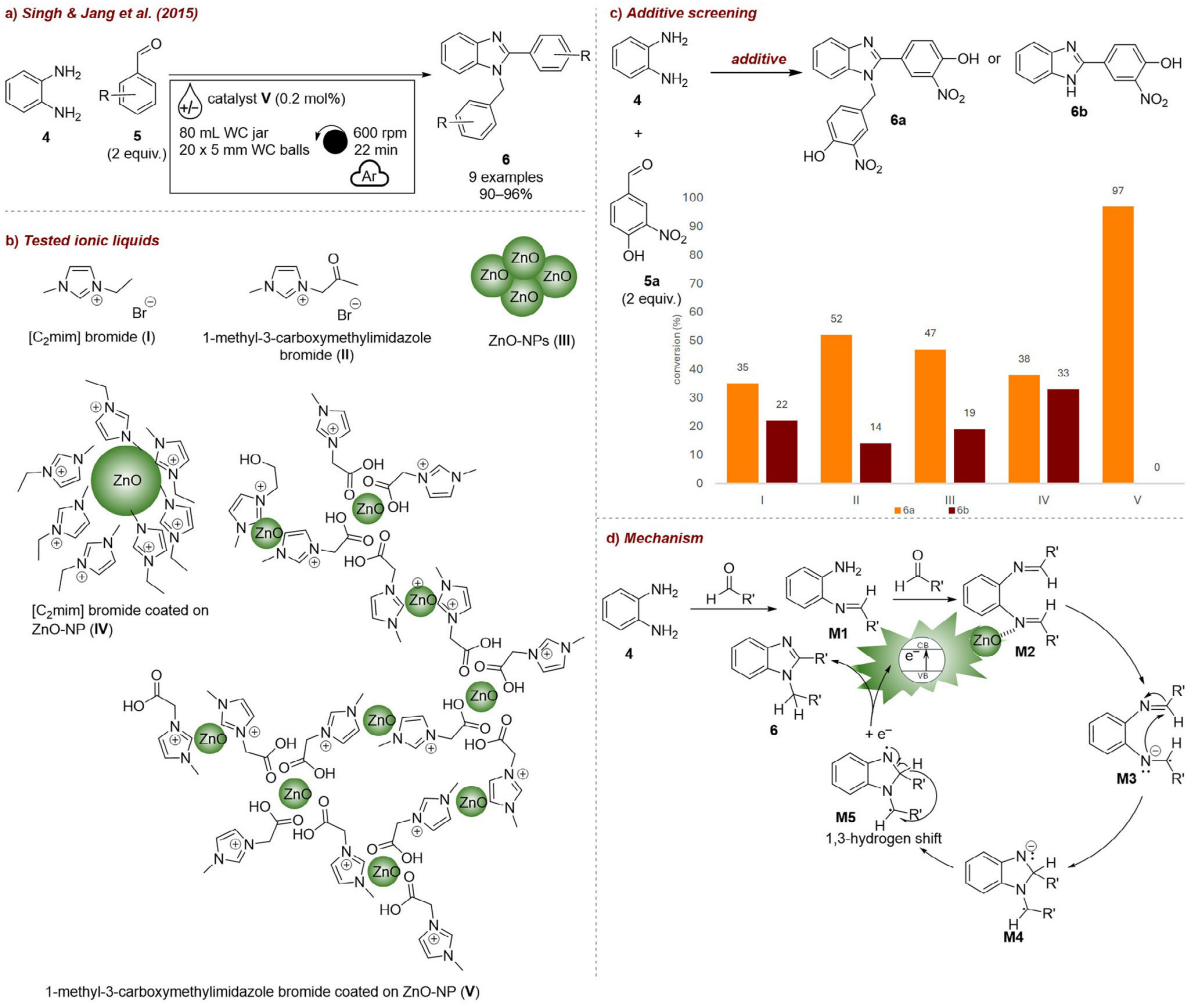
(a) Mechanochemical synthesis of 1,2‐disubstituted benzimidazoles **6** employing ILs. (b) Structures of tested IL additives. (c) Additive screening. Reaction conditions: **4** (9.2 mmol), **5a** (2 equiv.), *additive* (0.2 mol%), WC jar (80 mL), 30 × 5 mm WC balls, under argon atmosphere, planetary mill: 600 rpm, 22 min.

In 2015, Singh and Jang et al. [53] reported the first example of ILs serving as catalysts in mechanochemical synthesis. They achieved a solvent‐free selective synthesis of 1,2‐disubstituted benzimidazoles in a planetary ball mill (Figure 3a). Among the catalysts tested, [C_2_mim] bromide **I** (Figure 3b), and 1‐methyl‐3‐carboxymethylimidazole bromide **II** alone gave moderate yields (35% and 52%, respectively, Figure 3c), ZnO nanoparticles (NPs) **III** gave a similar outcome as catalyst **II**, and 1‐ethyl‐3‐methylimidazolium bromide coated on ZnO‐NPs **IV** offered no improvement. Finally, 1‐methyl‐3‐carboxymethylimidazole bromide coated on ZnO‐NPs **V** afforded only the desired product **6a**, with no byproducts detected. The efficiency of catalyst **V** was attributed to the synergistic effect of IL **II** and ZnO‐NPs **III**. ZnO‐NPs have a strong affinity for COOH, OH, and CN groups, while ILs act as structure‐directing agents. In this case, a three‐dimensional network is formed between ZnO‐NPs **III** and IL **II** via the COOH linker group. Catalyst loading studies showed that 0.2 mol% was optimal, and productivity was found to be proportional to the ball‐to‐powder ratio, due to the corresponding increase in collision frequency. Mechanistic studies suggested that the mechanical energy from milling promotes electron excitation from the valence to the conduction band of ZnO. The resulting conduction‐band electron is transferred to the imine intermediate, generating an anionic radical **M3**, which is converted into a radical species **M4** that undergoes a 1,3‐hydrogen shift to give the final product. The reactions were performed on a 9.2 mmol scale and successfully scaled up to 80 mmol. Simple isolation of products by washing with water and efficient catalyst recyclability (up to six times) contributed to an exceptionally low E‐factor [54] of 0.17, the lowest for this class of reactions.

**FIGURE 4:**
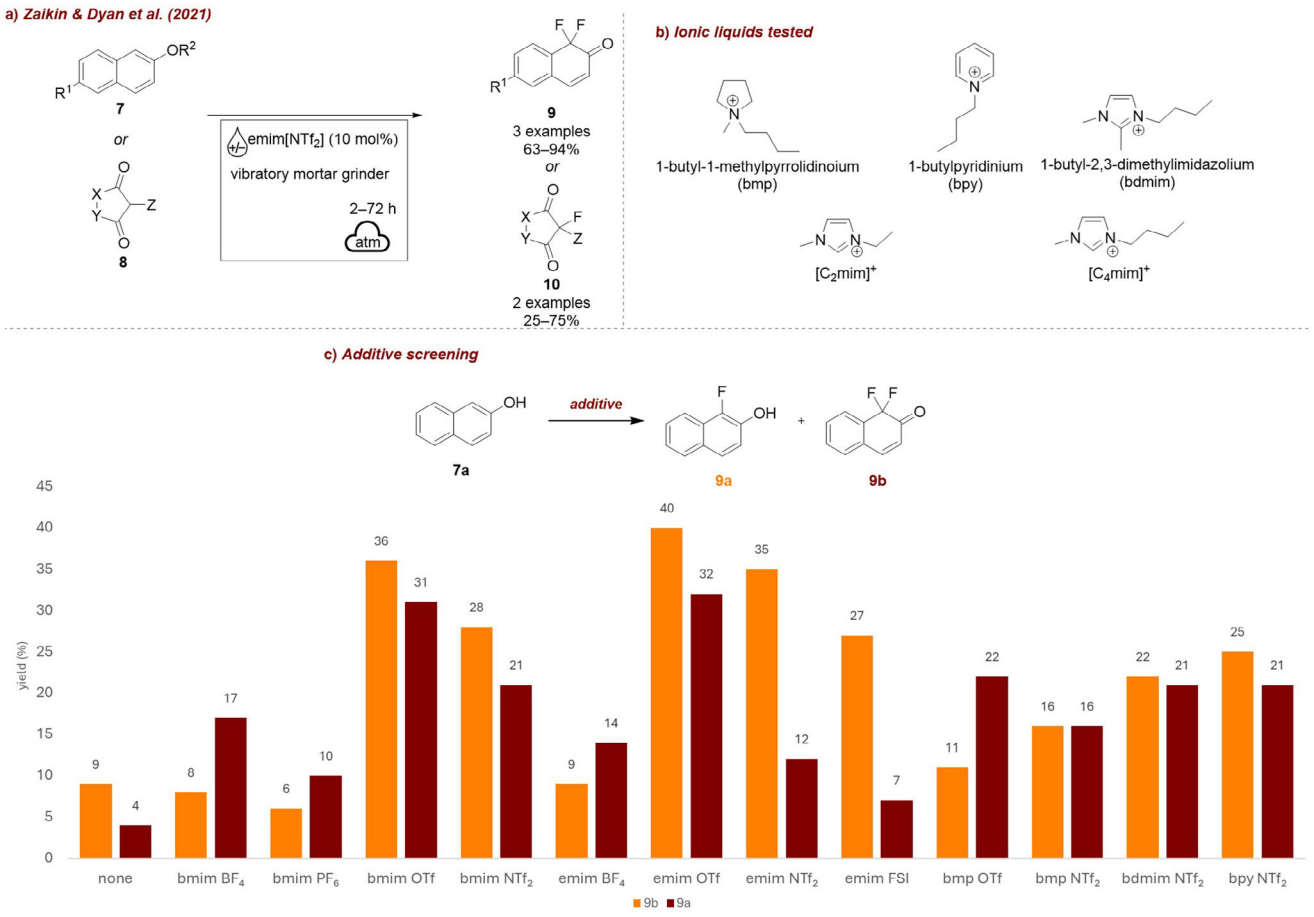
(a) Mechanochemical electrophilic fluorination of aromatic substrates **7** and 1,3‐dicarbonyl compounds **8** with selectfluor employing ILs. (b) Structures of tested ILs. (c) Additive screening. Reaction conditions: **7a** (1 mmol), selectfluor (2 equiv.), *additive* (10 mol%), 52 mm agate ball, vibratory mortar grinder: 2 h.

In the same year, Zaikin and Dyan et al. [55] established the first systematic comparison of various ILs as grinding additives in mechanochemical electrophilic fluorination. They performed electrophilic fluorination of aromatic substrates **7** and 1,3‐dicarbonyl compounds **8** (Figure 4a) with selectfluor in a vibratory mortar grinder. Among the modified naphthol derivatives was naproxen, a widely used nonsteroidal anti‐inflammatory drug, demonstrating that this method can also be applied to late‐stage modifications. A combination of ionic liquid‐assisted grinding (IL‐AG) with a vacuum sublimation of the product [56] enabled efficient isolation and afforded an E‐factor of 3, the lowest in its respective class of transformations. To investigate the influence of ILs on the reaction outcome, they tested bmp, bpy, and bdmim, [C_2_mim], and [C_4_mim] cations (Figure 4b) and BF_4_, PF_6_, OTf, NTf_2_, and fluorosulfonimide (FSI) anions. Even though no strict correlation was established, the authors found that ILs with lower viscosity generally gave higher conversions of both the substrate and the fluorinating reagent.

### 1.3 Ionic Additives

#### 1.3.1 Sodium Chloride (NaCl) as Main Additive

Sodium chloride (NaCl) is one of the most abundant and inexpensive inorganic salts, readily available worldwide as table salt or industrial‐grade mineral halite. Its low cost and wide availability make it attractive for large‐scale applications. Chemically, sodium chloride is highly stable and inert under most reaction conditions, which ensures it does not interfere with mechanochemical transformations. It is also nontoxic and easy to handle [57].

In most cases, NaCl serves a purely physical purpose as bulking agent or solid support, without directly participating in the chemical transformation. Although chemically inert in these contexts, it is often essential for reproducibility and scalability in mechanochemical processes. In this review, we decided to highlight some noteworthy examples.

**FIGURE 5:**
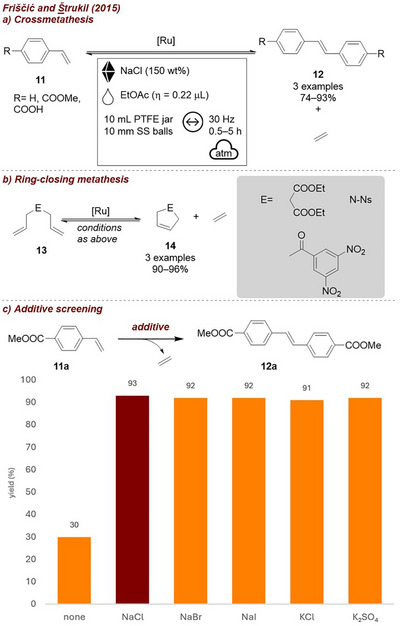
(a) Mechanochemical Ru‐catalyzed olefin cross‐metathesis. (b) Mechanochemical Ru‐catalyzed ring‐closing metathesis. (c) Additive screening. Reaction conditions: **11a** (2 mmol), Hoveyda–Grubbs second generation (1 mol%), *additive* (225 mg/mmol), PTFE jar (10 mL), 1 × SS ball (10 mm), mixer mill: 30 Hz, 1.5 h.

In 2015, Friščić and Štrukil [23] developed a mechanochemical approach for Ru‐catalyzed olefin and ring‐closing metathesis applicable to both liquid and solid olefins (Figure 5a, b). While liquid substrates underwent metathesis readily, solid olefins required the use of a combination of LAG agent along with a solid auxiliary to achieve high and reproducible yields (Figure 5c). The specific nature of the salt auxiliary showed little impact on the outcome, as all tested solids gave similar results, confirming that the additive served purely as a bulking agent. However, the solid additive was still essential to achieve high yields. Notably, both cross‐metathesis and ring‐closing metathesis were attainable on a gram scale with reproducible yield.

**FIGURE 6:**
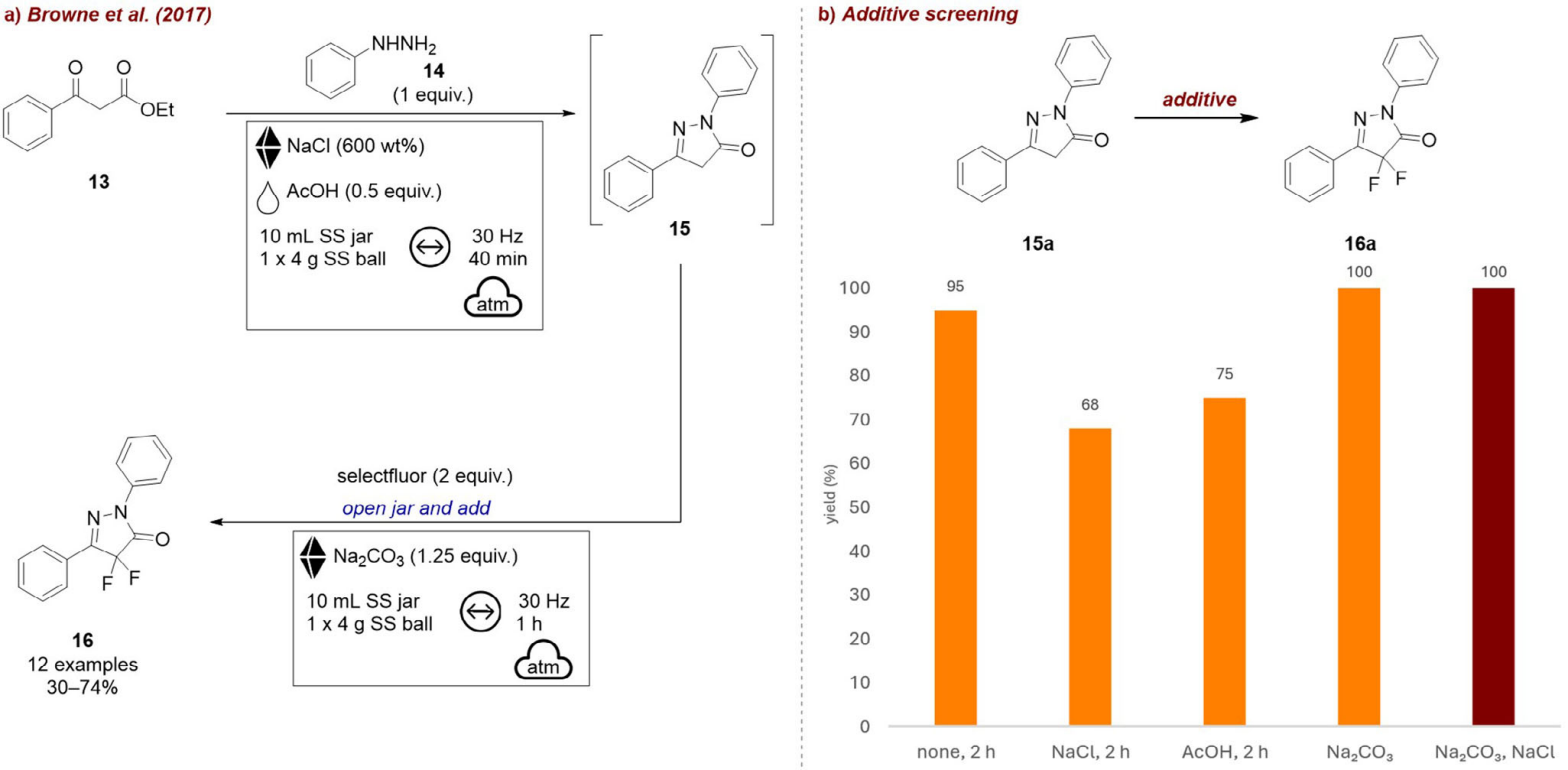
(a) Two‐step, one‐jar process for heterocycle formation and subsequent fluorination of pyrazolones. (b) Additive screening. Reaction conditions: **15a** (1 equiv.), selectfluor (2 equiv.), *additive*, SS jar (10 mL), 1 x SS ball (4 g), mixer mill: 30 Hz, 1 h. Yield determined by ^19^F‐NMR.

In 2017, the Browne group [58] reported a two‐step, one‐jar process for heterocycle formation and subsequent fluorination of pyrazolones (Figure 6a). Since both reagents **13** and **14** in the initial step were liquids, NaCl was employed as a grinding auxiliary. Additionally, to catalyze pyrazolone **15** formation, 0.5 equivalents of acetic acid (AcOH) were added; however, both the auxiliary and acetic acid, respectively, were found to slow down the fluorination step (Figure 6b). As a workaround, the addition of sodium carbonate (Na_2_CO_3_) in the second step, resulted in complete conversion after 1 h. Moreover, if Na_2_CO_3_ was employed NaCl did not diminish reaction efficiency anymore. These final conditions balance acid neutralization and reaction acceleration, allowing a one‐pot process toward products **16**.

**FIGURE 7:**
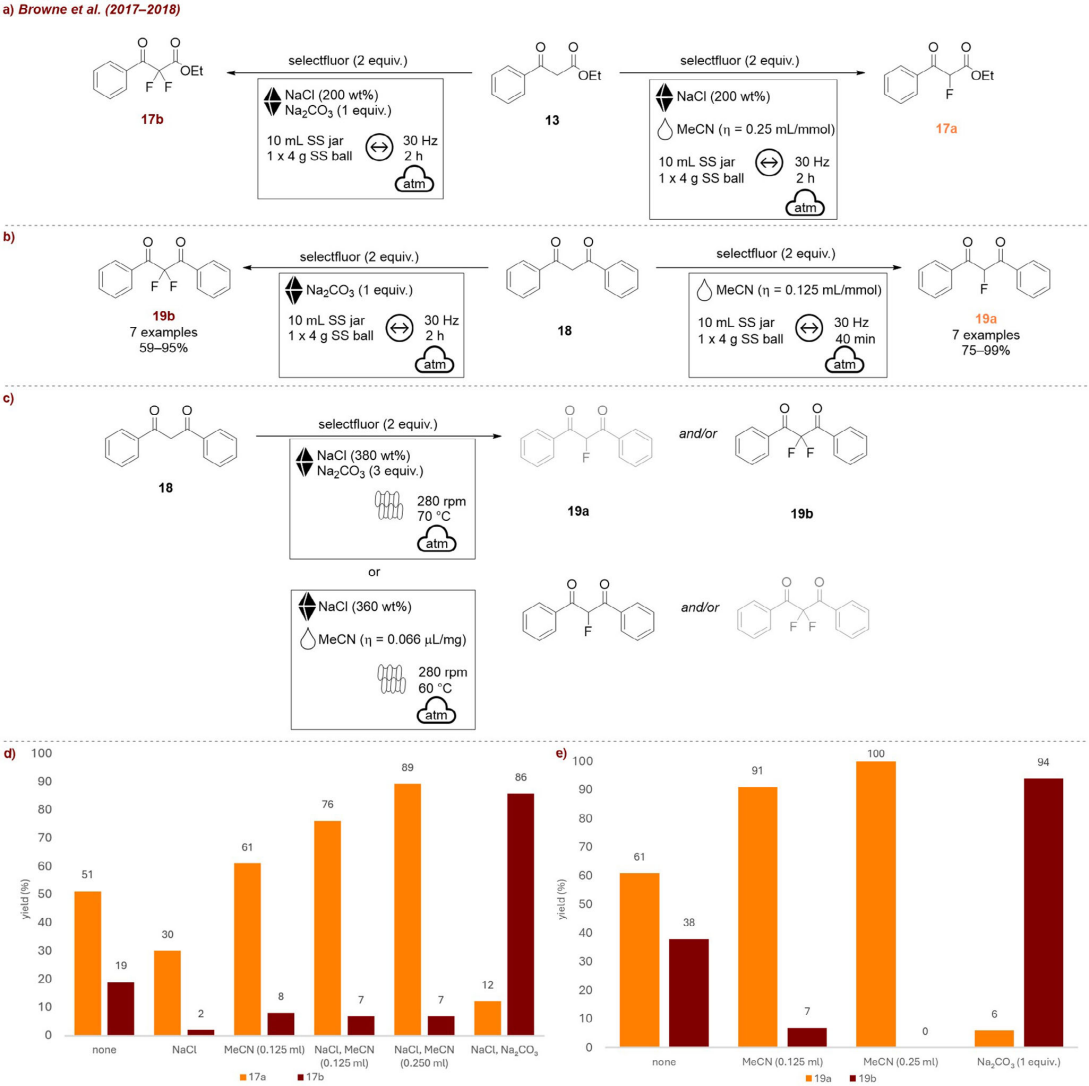
(a) Mono‐ and di‐fluorination of carbonyls. (b) Mono‐ and di‐fluorination of diketones. (c) Continuous extrusion of mono‐ and di‐fluorination of diketones. (d) Screening conditions: **13** (1 equiv.), selectfluor (2 equiv.), *additive* (NaCl‐200 wt%, Na_2_CO_3 –_ 1 equiv.), SS jar (10 mL), 1 × SS ball (4 g), mixer mill: 30 Hz, 2 h. Yield determined by ^19^F‐NMR. (e) Screening conditions: **18** (1 mmol, 1 equiv.), selectfluor (2 equiv.), *additive*, SS jar (10 mL), 1 × SS ball (4 g), mixer mill: 30 Hz, 2 h. Yield determined by ^19^F‐NMR.

Soon after, the same group reported the mechanochemical electrophilic fluorination of dicarbonyls **13** [9] (Figure 7a). In this work they demonstrate that selectivity between mono‐ **17a** and difluorinated β‐ketoesters **17b** could effectively be tuned by choice of additive (Figure 7d). In their screening studies, they found that the sole addition of NaCl as grinding agent diminished the overall yield. While MeCN on its own had no effect on yield (remains at around 70%), selectivity for the product **17a** improved (7.6:1 compared to 2.7:1). When both MeCN and NaCl were used together, the yield increased significantly, with **17a** being formed predominantly. In contrast, using Na_2_CO_3_ in combination with NaCl (without MeCN) allowed the selective formation of the di‐fluorinated product **17b**. This work is an excellent example for how strategic variation of additives allows control of chemoselectivity in mechanochemical transformations. Accordingly, in a different report [38], they described the selective fluorination of 1,3‐diketones **18**, once more demonstrating the influence of liquid additives on reaction outcome (Figure 7b). Addition of various additives increased reaction efficiency, as well as product selectivity (Figure 7e). Comparable to their other study, MeCN as LAG‐agent led to the selective formation of the monofluorinated product **19a**, while omitting liquid additives and using Na_2_CO_3_ favored the difluorinated product **19b**. A few years later, they transitioned this reaction from the mixer mill to a continuous twin‐screw extruder to develop a scalable method for difluorination to afford product **19b** in a continuous process [59] (Figure 7c). A combination of Na_2_CO_3_ and NaCl was found to be optimal, with the grinding auxiliary playing a crucial role in preventing agglomeration, as increasing the amount relative to the reactant, formation of hard clumps could be prevented. Additionally, the addition of a liquid additive enabled selective access to the mono‐fluorinated product **19a**, demonstrating that the extrusion conditions could be tuned to control selectivity once more. It should be noted that, while it is possible that it additionally acts as grinding auxiliary, the purpose of Na_2_CO_3_ is to act as a base. Still, these reactions show that different outcomes can be achieved by using a solid, inert additive (NaCl), and/or LAG.

**FIGURE 8:**
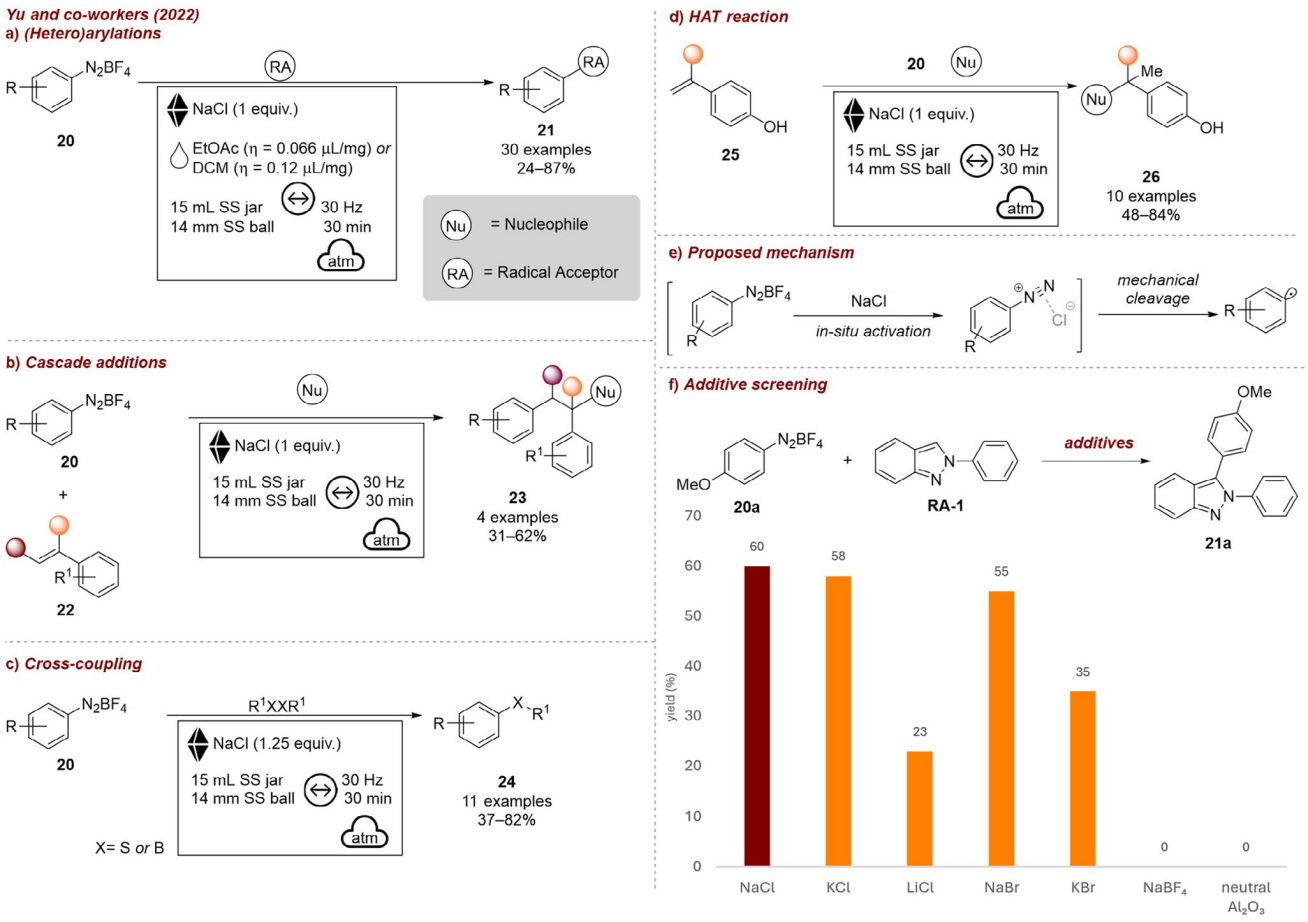
(a) Mechanochemical (hetero)arylation reaction. (b) Mechanochemical cascade additions. (c) Mechanochemical cross‐coupling reactions. (d) Mechanochemical HAT reactions. (e) Proposed mechanism for mechanochemical generation of aryl radical from aryldiazonium salts. (f) Additive screening. Reaction conditions: **20** (2.0 equiv.), **RA‐1** (0.3 mmol, 1.0 equiv.) *additive* (1.0 g), EtOAc (𝜂  =  0.051 µL/mg), SS vessel (15 mL), 1 × SS ball (14 mm), mixer mill: 30 Hz, 30 min.

A noteworthy example, in which the ionic additive serves as both grinding aid and activator, was reported by Yu and co‐workers in 2022 [60]. They described the mechanochemical generation of an aryl radical from aryldiazonium salts **20**, enabling various radical transformations such as (hetero)arylations (Figure 8a), cascade additions (Figure 8b), cross‐couplings (Figure 8c), and hydrogen atom transfer reactions (Figure 8d), employing NaCl. For these reactions, they propose that the noncoordinating tetrafluoroborate counter ion of the diazonium salt is exchanged by the chloride promoter ion. Upon mechanical stress, homolysis is triggered, liberating nitrogen and forming an aryl radical, thus enabling numerous transformations (Figure 8e). Therefore, the type of grinding auxiliary showed to have a major influence on reaction outcome (Figure 8f). During optimization of the arylation reaction, various additives were tested. Most halogen salts (NaCl, NaBr, KCl) gave similar results, while nonhalogen containing salts, such as Al_2_O_3_ and NaBF_4_, gave no products, supporting the proposed mechanism. Salts with weaker ionic character (LiCl, KBr) afforded the product in only moderate yields. Reactions without a solid additive afforded no products. As for the heteroarylation reaction, where both starting materials were of solid nature, the authors additionally investigated liquid additives to increase dissolution and diffusion efficiency. A small amount of ethyl acetate (𝜂  =  0.066 µL/mg) was found to increase the yield from 60% to 84%. Remarkably, no specific purity for the solid additive was required and NaCl could be recycled five times without loss of activity.

**FIGURE 9:**
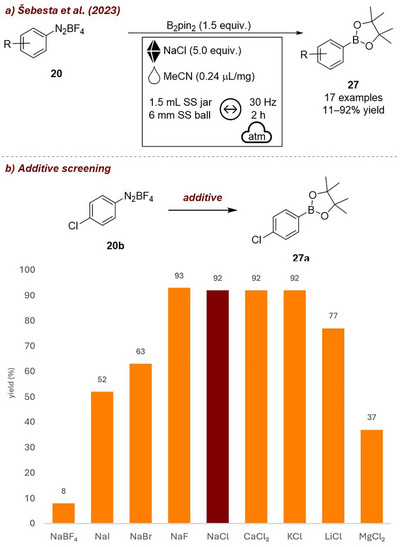
(a) NaCl‐induced radical borylation of diazonium salts. (b) Additive screening. Reaction conditions: **20b** (0.3 mmol), B_2_pin_2_ (1.5 equiv.), *additive* (5 equiv.), MeCN (0.24 µL/mg), SS vessel (1.5 mL), 1 × SS ball (6 mm), mixer mill: 30 Hz, 2 h. Yield determined by GC‐FID analysis.

Concurrently, Šebesta et al. [61] explored a related mechanochemical process, namely the NaCl‐induced radical borylation of aryl diazonium salts **20** (Figure 9a). Their detailed investigation on the impact of various inorganic salts on the borylaton of compound **20b** emphasized the critical role of the ionic character of the additive in facilitating the key ion exchange step. Highly ionic salts, such as NaF, NaCl, CaCl_2_, KCl, consistently led to excellent results (92%–93% yield, Figure 9b). Salts with more covalent character were significantly less effective, highlighting the direct influence of the nature of the additive on reactivity through its impact on ion‐exchange efficiency. This supports the proposed ion exchange leading to a homolytic scission mechanism. They also investigated different LAG‐agents, observing that the best results were achieved using acetone, ethyl acetate, or ethanol (92%–99%), probably as these achieve optimal mixing of NaCl and the diazonium salts.

**FIGURE 10:**
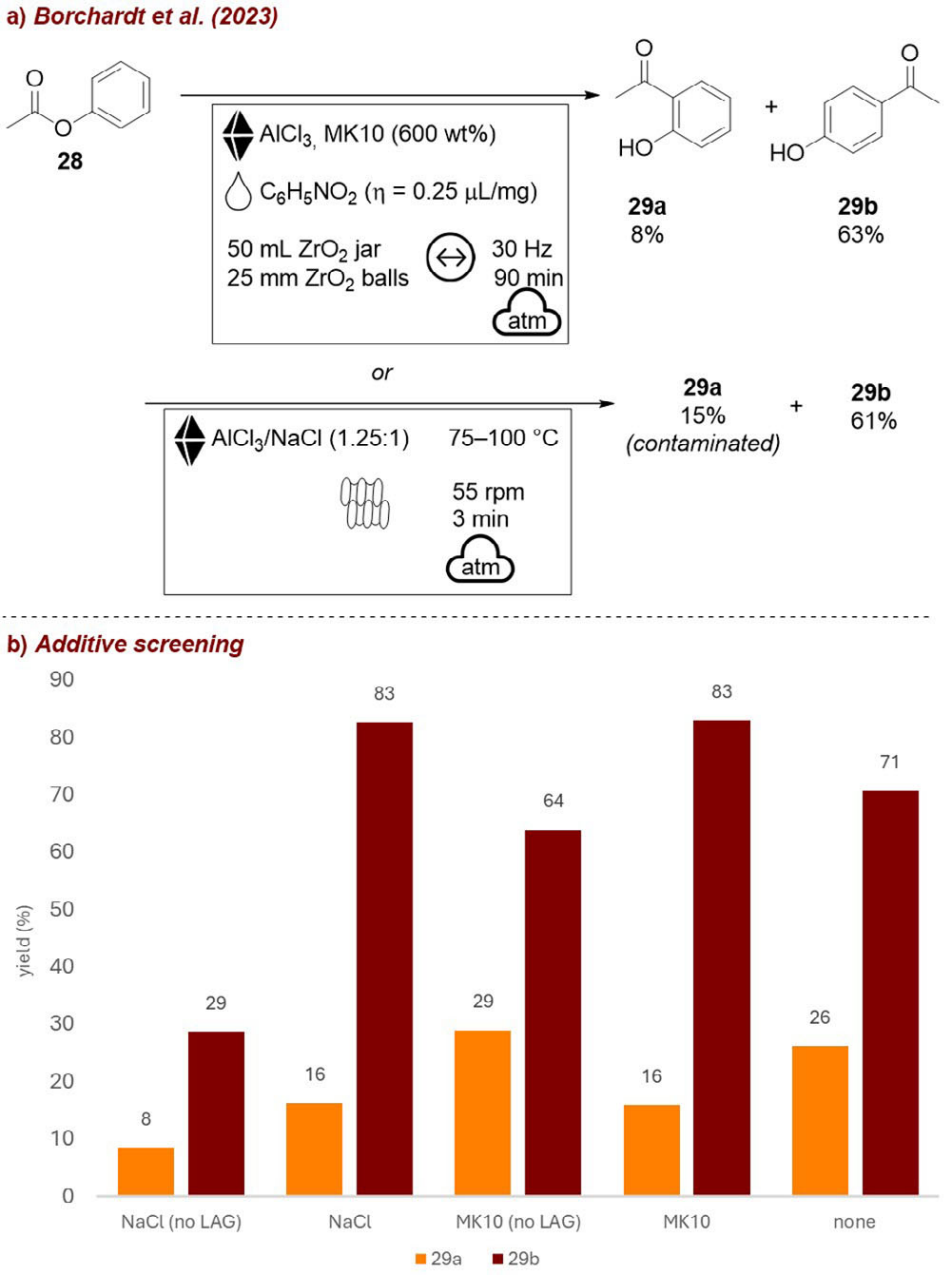
(a) Mechanochemical Fries rearrangement in a ball mill and SS extruder employing NaCl as solid additive. (b) Additive screening. Reaction conditions: **28** (1 mL), AlCl_3_ (3.14 g), *additive*, nitrobenzene (LAG, 𝜂  =  0.25 µL/mg), NaCl (2.5 g) or MK10 (6.12 g), ZrO_2_ jar (50 mL), ZrO_2_ balls (25 mm), mixer mill: 30 Hz, 90 min. Conversion/selectivity determined by GC‐MS.

More recently, the Borchardt group [62] employed NaCl and Montmorillonite K10 (MK10) as solid additives to improve reaction efficiency in the mechanochemical Fries rearrangement (Figure 10a). It was found that different liquid additives or bulking materials led to different *para/ortho*‐ratios (Figure 10b). No solid additive afforded a mixture of products with a 2.7 ratio (**29b** to **29a**) with high conversion (97%). Employing a combination of nitrobenzene as LAG‐agent and MK10 or NaCl, respectively, led to similar results (99% yield, 5.1–5.2 **29b**/**29a** ratio). Without the liquid additive, the reaction led to diminished yields and a lower *para*‐selectivity. While in mixer mill experiments, MK10 was essential to avoid caking and ensure high conversion, NaCl was used in TSE to serve a similar bulking and grinding function despite its tendency to cause agglomeration in milling experiments. LAG in the extruder had no positive influence, as sufficient mixing was achieved by the extruder itself.

### 1.4 Alumina (Al_2_O_3_) as Main Additive

Alumina exhibits ion‐exchange behavior due to its amphoteric nature, which depends on the pH of the surrounding solution. This property arises from two types of surface hydroxyl groups: hydroxyls chemisorbed onto aluminum atoms and protons chemisorbed onto oxygen atoms. When neutral alumina is treated with sodium hydroxide, the chemisorbed protons are replaced by sodium ions, enabling cation‐exchange behavior at higher pH. Conversely, washing alumina with hydrochloric acid introduces protons that displace hydroxyl groups and sodium ions, allowing chloride ions to bind and impart anion‐exchange properties under acidic conditions [63].

**FIGURE 11:**
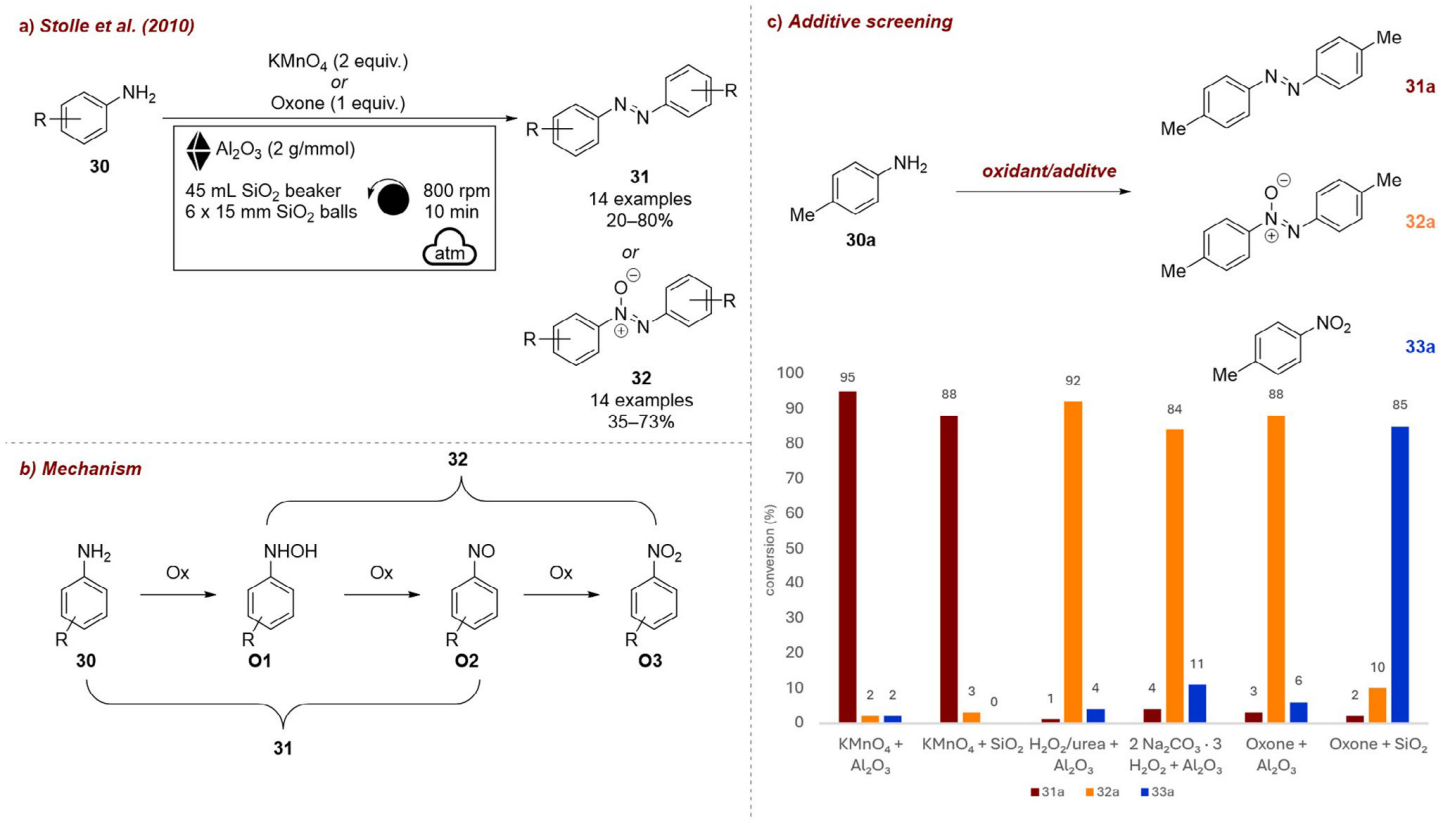
(a) Mechanochemical oxidation of anilines to the corresponding azo‐ and azoxy‐homocoupling products in the planetary ball mill. (b) Additive screening. Reaction conditions: **30a** (2 mmol, 1 equiv.), *oxidant* (2 equiv.), *additive (*4 g), agate beaker (45 mL), 6 × agate ball (15 mm), planetary mill: 800 rpm, 10 min. Conversion determined by GC‐FID.

Stolle and co‐workers [64] reported a solvent‐free method for the oxidation of anilines **30** to the corresponding azo‐ **31** and azoxy‐homocoupling products **32** in a planetary ball mill (Figure 11a). This reaction proceeds via the partial oxidation of anlines to either hydroxylamines **O1** or nitroso‐compounds **O2**, which upon condensation afford the azo or azoxy products, respectively. By varying the combination of oxidant and grinding auxiliary, they were able to control product selectivity (Figure 11c). KMnO_4_ in combination with neutral alumina or silica favored the formation of the azo‐product **31a**, whereas the azoxy products **32a** were preferentially obtained using alumina with either oxone, H_2_O_2_/urea, or sodium percarbonate. Notably, when alumina was replaced by silica and oxone was used as oxidant, the nitro‐product **33a** was selectively accessible.

**FIGURE 12:**
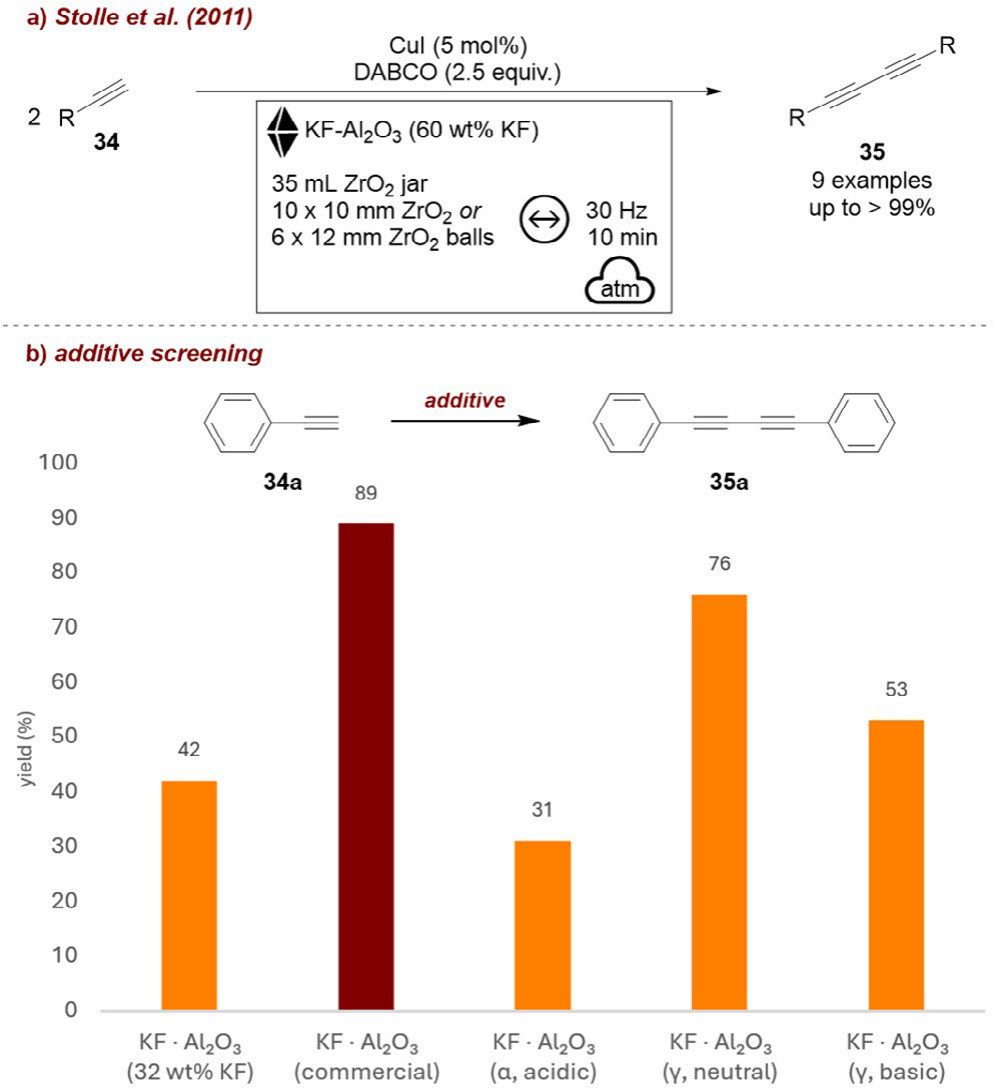
(a) Mechanochemical Glaser reaction—homocoupling of terminal alkynes—employing KF‐Al_2_O_3_ as milling auxiliary. (b) Additive screening. Reaction conditions: **34a** (2 mmol, 1 equiv.), CuI (5 mol%), *additive system* (40 wt% KF on Al_2_O_3_, 4 g), ZrO_2_ beaker (35 mL), 12 × ZrO_2_ ball (10 mm), mixer mill: 30 Hz, 10 min. Yield determined by GC‐FID.

The same authors also investigated the Glaser reaction [65], the homocoupling of terminal alkynes **34**, where they used KF‐Al_2_O_3_ as milling auxiliary, which simultaneously serves as base (Figure 12a). It was shown that changes in the modifications of the alumina type (acidic/basic/neutral) had an impact on the homocoupling of phenylacetylene **34a**, the commercially available, neutral Al_2_O_3_ performing best (Figure 12b). Furthermore, the KF content within the KF–Al_2_O_3_ system significantly impacted the reaction yield, a higher KF‐content reaching higher yields of **35a**. When 60 wt% KF on neutral Al_2_O_3_ were used, the reaction proceeded without the need for an external base, reaching almost quantitative yield. They proceeded their study with the KF‐Al_2_O_3_ (60 wt% KF) prepared by an impregnation method, where neutral Al_2_O_3_ is dissolved in an aqueous KF‐solution and subsequent removal of solvent, achieving excellent yields of up to >99% for 9 examples. A related strategy had already been applied by the same group [66] in the Suzuki–Miyaura transformation, where KF/Al_2_O_3_ was used, not as a milling auxiliary, but as a solid support for the in situ generation of the base required for the coupling.

**FIGURE 13:**
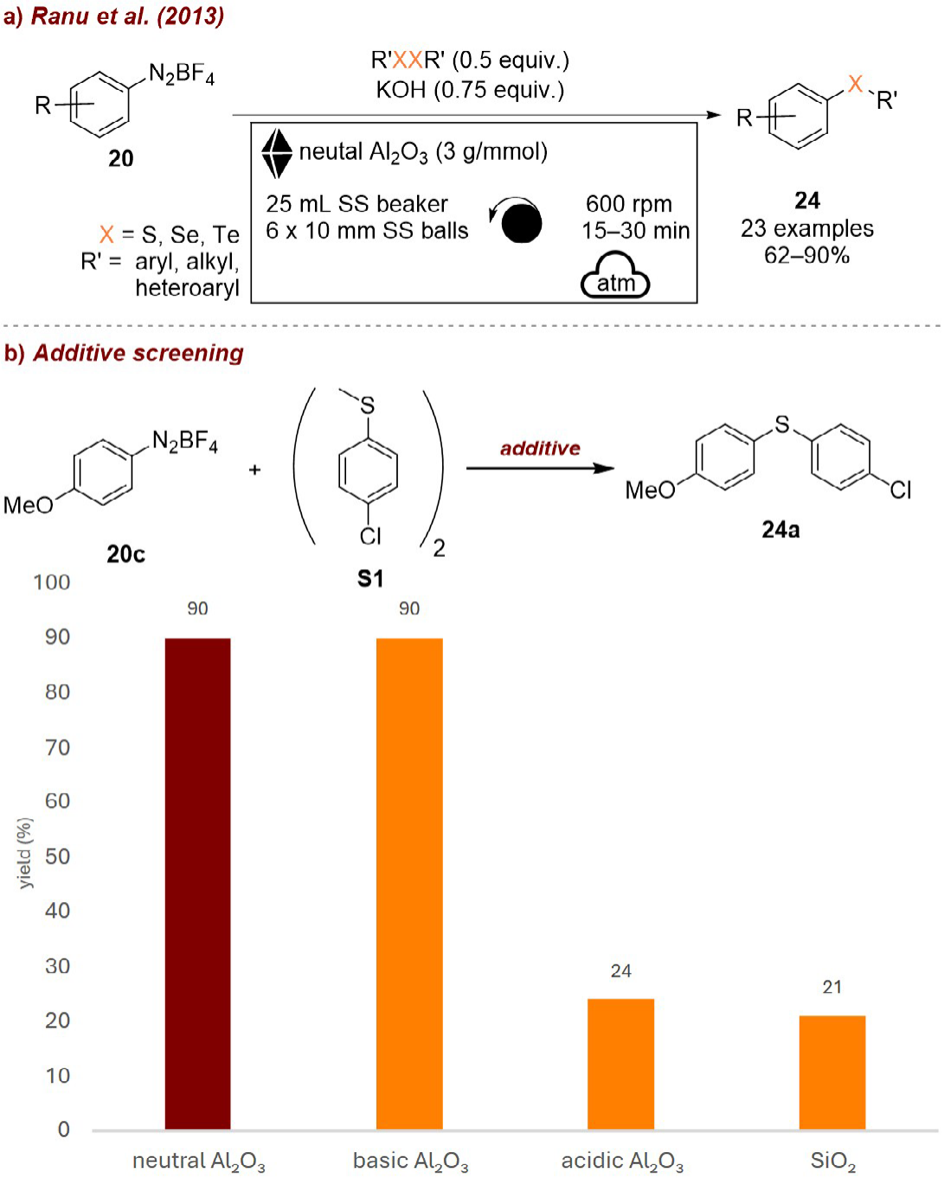
(a) Mechanochemical synthesis of diaryl chalcogenides from diazonium tetrafluoroborates. (b) Additive screening. Reaction conditions: **20c** (1 mmol), **S1** (0.5 equiv.), KOH (0.75 equiv.), *additive* (3 g), SS beaker (25 mL), 6 × SS balls (10 mm), planetary mill: 600 rpm, 15 min. Yield determined by GC‐FID analysis.

Another mechanochemical study involving diazonium tetrafluoroborates **20** was published by Ranu et al. in 2013 [67], presenting a general procedure for the synthesis of diaryl chalcogenides **24** (Figure 13a). The authors investigated all three types of alumina (basic, neutral, and acidic) as well as silica gel as grinding auxiliary for the reaction of **20c** with sulfane **S1** and found that neutral and basic alumina significantly outperformed the others, affording **24a** in 90% (Figure 13b). While they offer no explanation for these results it is reasonable to assume that the acidic character of silica and the acidic modification of alumina partially neutralize the base required for the reaction, thereby leading to a decrease in overall reaction efficiency. This rational is in accordance with their results for reactions performed with lower amounts of base affording lower yields (83% for 0.5 equiv. KOH).

**FIGURE 14:**
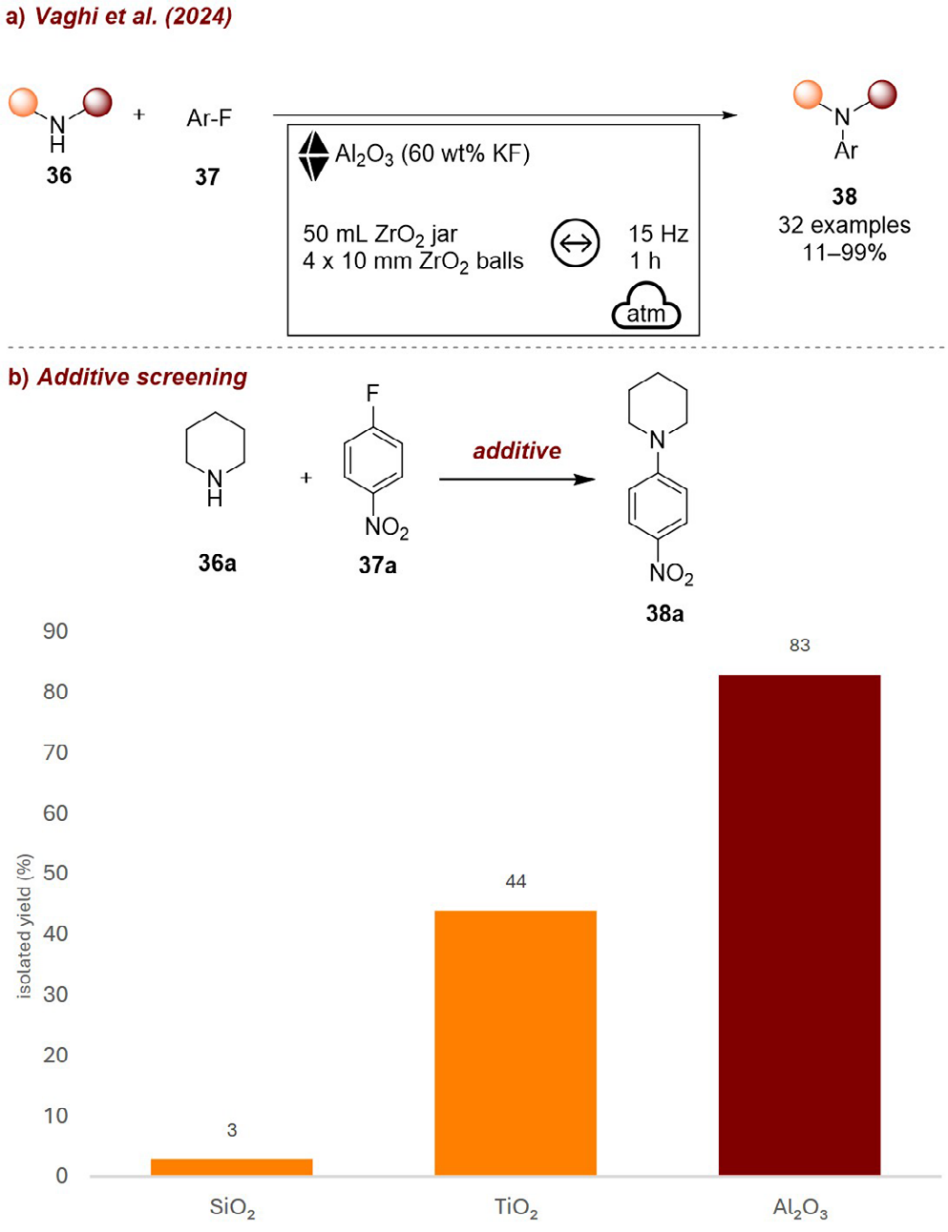
(a) Mechanochemical nucleophilic aromatic substitution of aryl fluorides with secondary amines. (b) Additive screening. Reaction conditions: **36a** (2 mmol, 1 equiv.), **37a** (2 mmol, 1 equiv.), *additive* (5 g), ZrO_2_ beaker (50 mL), 4 × ZrO_2_ balls (10 mm), mixer mill: 15 Hz, 60 min.

Another example where the additive functions as both a milling auxiliary and promoter was reported by Vaghi et al. [68] in 2024 (Figure 14a). In their study investigating the mechanochemical nucleophilic aromatic substitution between secondary amines **36** and aryl‐fluorides **37**, the use of Al_2_O_3_ as grinding auxiliary led to exceptional results without the need for additional base (Figure 14b). This effect was attributed to the optimal HF affinity of Al_2_O_3_, which in turn prevents protonation of the remaining nucleophile, and thereby sustaining the reaction efficiency. Other oxides like silica or TiO_2_ afforded the product in only low‐to‐moderate yields. This protocol allowed the preparation of a variety of amino substituted aryls and heteroaryls **38** in moderate‐to‐excellent yields.

### 1.5 Other Ionic Salts

**FIGURE 15:**
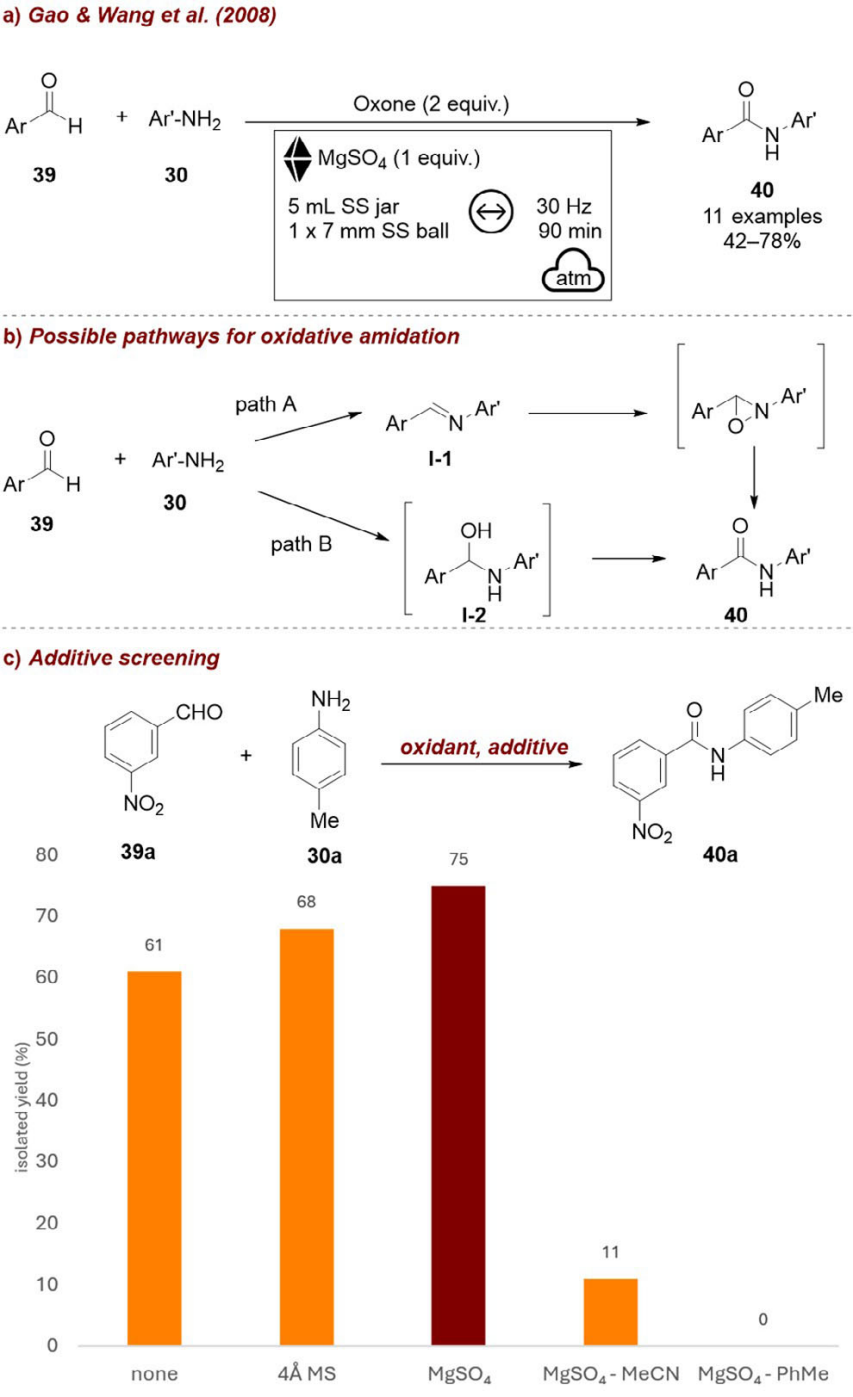
(a) Mechanochemical one‐pot approach for the oxidative amidation of aldehydes with anilines, using oxone as the oxidant. (b) Possible pathways for oxidative amidation. (c) Additive screening. Reaction conditions: solvent‐free: **39a** (1 equiv.), **30a** (1 equiv.), oxone (2 equiv.), *additive*, SS jar (5 mL), SS ball (7 mm), mixer mill: 30 Hz, 90 min. In solution: **39a** (1 equiv.), **30a** (1 equiv.), MgSO_4_ (1 equiv.) in solvent (MeCN/PhMe). MS = molecular sieves.

In an example from 2008, Gao and Wang [69] reported a mechanochemical one‐pot approach for the oxidative amidation of aldehydes **39** with anilines **30**, using oxone as the oxidant (Figure 15a). As one proposed reaction pathway likely involves an imine intermediate **I‐1** (Figure 15b), which would generate water and thus negatively affect the reaction, they investigated various additives to remove it (Figure 15c). Pleasingly, the addition of water scavengers, molecular sieves, and anhydrous magnesium sulfate (MgSO_4_) enhanced the reaction efficiency, simultaneously supporting the proposed mechanism. In particular, for the reaction of aldehyde **39a** and aniline **30a**, MgSO_4_ afforded the desired amide **40a** in 75% yield. Moreover, performing the reaction in toluene or MeCN resulted in markedly diminished efficiency relative to the solvent‐free protocol, underscoring the utility of the latter.

**FIGURE 16:**
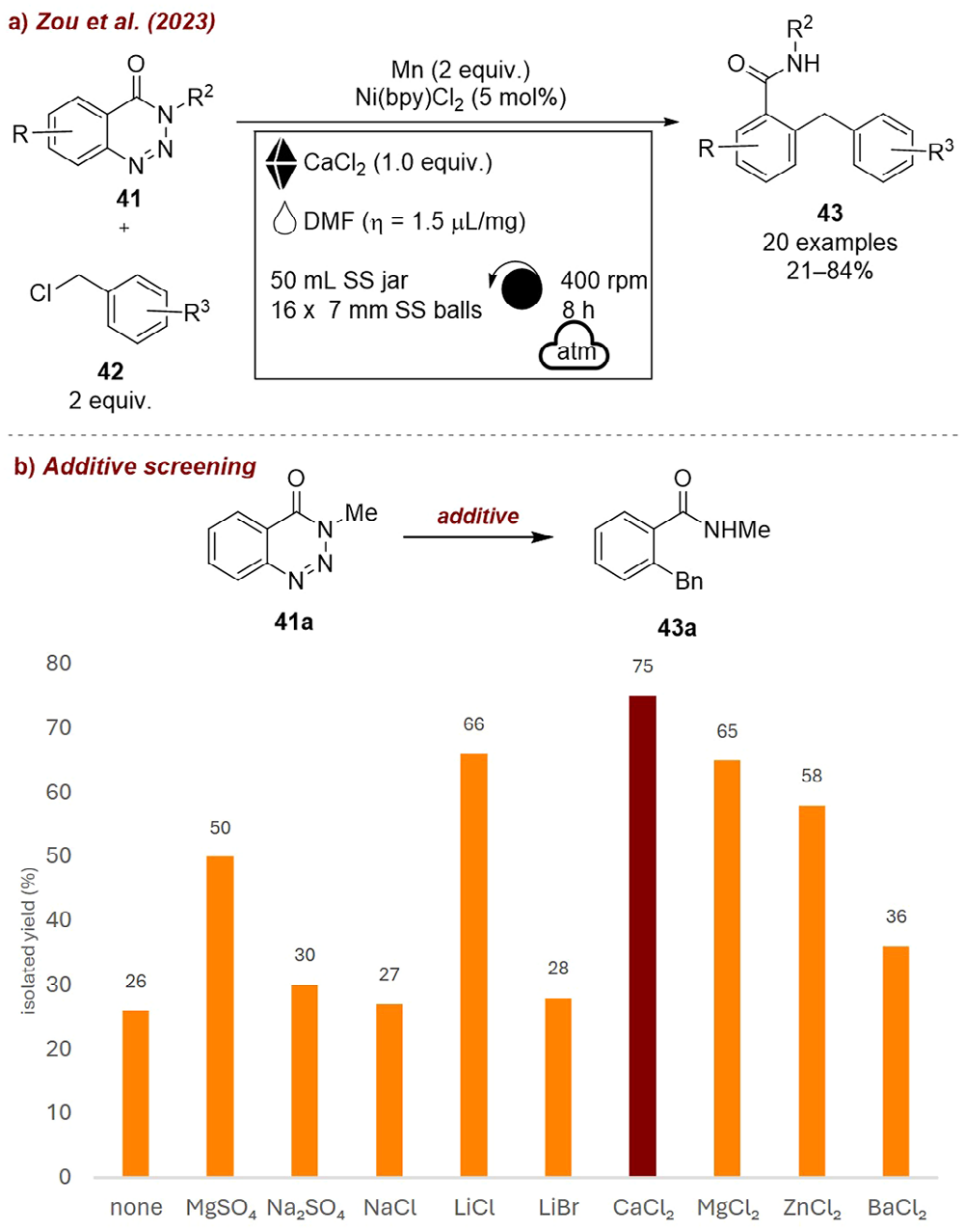
(a) Mechanochemical Ni‐catalyzed, air‐tolerant denitrogenative cross‐electrophile coupling of benzotriazinones with benzyl chlorides. (b) Additive screening. Reaction conditions: **41a** (2 mmol), **42** (1.5 equiv.), Ni(bpy)Cl_2_ (5 mol%), Mn powders (2 equiv.), DMF (𝜂  =  1 µL/mg), *additive (1* equiv*.)*, SS jar (50 mL), 16 × SS balls (7 mm), planetary mill: 400 rpm, 8 h.

In a very recent study, Zou et al. [14] reported a Ni‐catalyzed, air‐tolerant denitrogenative cross‐electrophile coupling of benzotriazinones **41** with benzyl chlorides **42** using dimethylformamide (DMF) as liquid additive (Figure 16a). These reactions typically require rigorously anhydrous conditions, which was true here as well. Among various water scavenging salts tested (Figure 16b), one equivalent of anhydrous calcium chloride (CaCl_2_) led to the best results, significantly outperforming salt‐free conditions, enabling the reaction to proceed efficiently under air‐tolerant but moisture sensitive conditions. It is noteworthy, in this example, DMF was essential as LAG‐additive. When other liquids were employed only traces of product were observed, suggesting that DMF plays a catalytic role in the reaction mechanism, namely facilitating the regeneration by reduction of Ni(II) to Ni(0).

**FIGURE 17:**
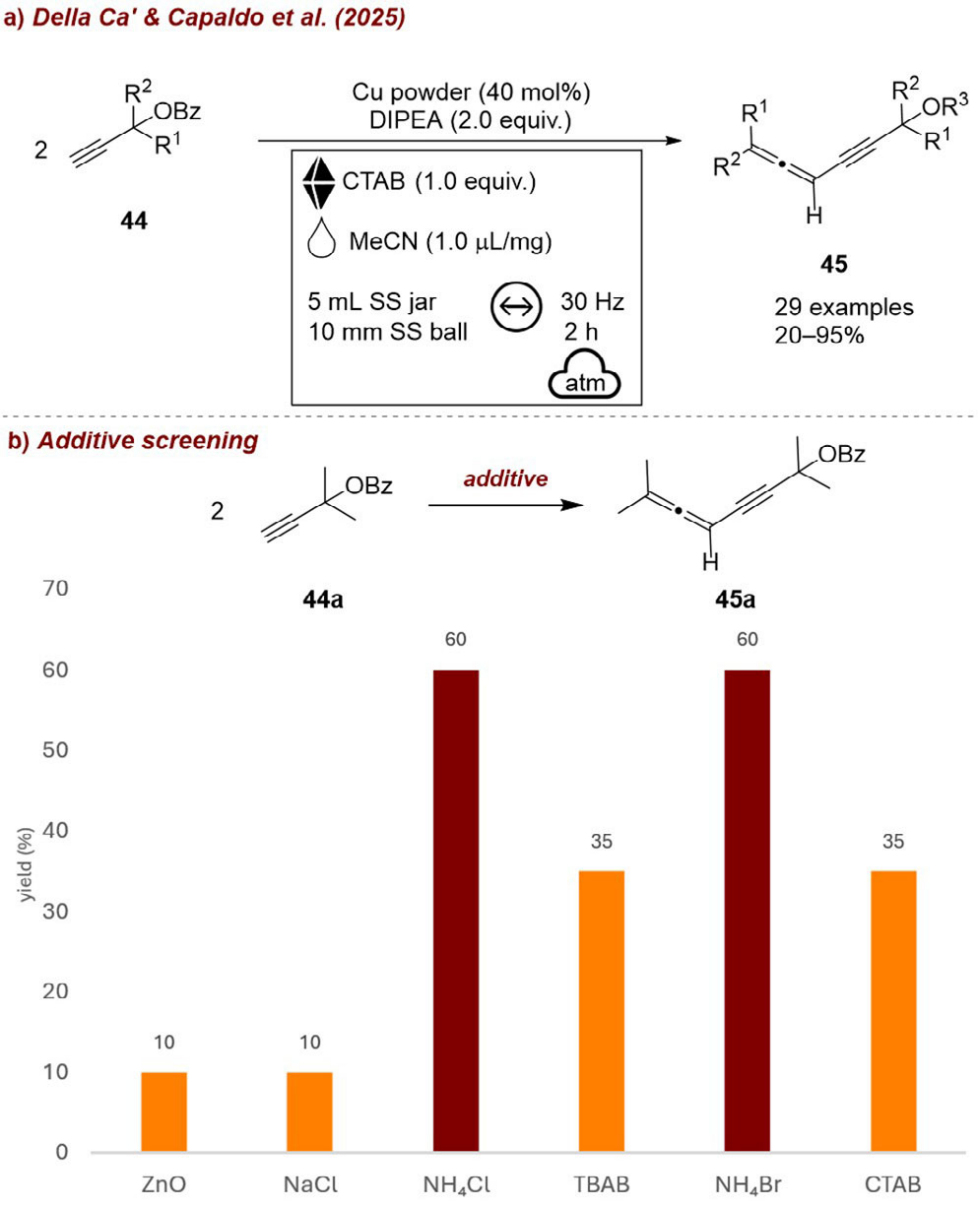
(a) Mechanochemical Cu(I)‐mediated homocoupling of two propargylic esters with CTAB as additive. (b) Additive screening. Reaction conditions: **44a** (0.2 mmol), Cu powder (40 mol%), DIPEA (0.2 mmol), *additive* (0.2 mmol), MeCN (𝜂  =  1.0 µL/mg), SS jar (5 mL), SS ball (10 mm), mixer mill: 30 Hz, 2 h. Yield determined by ^1^H NMR spectroscopy.

In one instance, Della Ca’, Capaldo, and co‐workers [70] demonstrated the Cu(I)‐mediated homocoupling of two propargylic esters **44**, proceeded via a copper–allenylidene intermediate to afford a conjugated allene product **45** (Figure 17a). The authors showed that, under ball‐milling conditions, metallic copper can be aerobically activated in situ to generate catalytically active oxidized copper species. Notably, the effect of various additives was investigated, and ammonium salts proved to be the most effective (Figure 17b). Under the final optimized conditions, cetyltrimethylammonium bromide (CTAB) was identified as the most efficient additive. The authors rationalized this behavior by proposing that the amine and halide components of the ammonium salt likely stabilize the in situ formed Cu(I) species as CuBr, enhancing its reactivity compared to metallic copper, which requires an induction period likely associated with the oxidation step.

**FIGURE 18:**
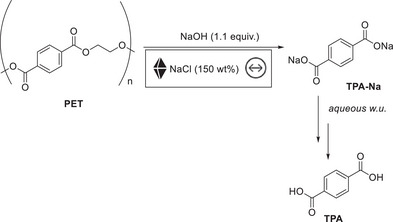
Additive‐assisted mechanochemical methods for environmental detoxification. Mechanochemical degradation of PET.

This review provides an overview of mechanochemical additive‐assisted synthesis of small organic molecules. The concepts presented within this work have also been applied to other fields in organic chemistry and chemical industry. The highly efficient mechanochemical hydrolysis of polyethylene terephthalate (PET) reported by Štrukil [71] in 2021 (Figure 18) highlights the industrial promise of solid‐state depolymerization. Conventional alkaline PET degradation requires harsh conditions, such as elevated temperatures and pressures, to obtain high conversion and terephthalic acid (TPA) yields. In the mechanochemical approach, waste PET is milled with NaOH (1.1 equiv.) together with NaCl (150 wt%) as grinding auxiliary. The additive was found to be crucial to ensure high reaction reproducibility and to facilitate the recovery of reaction products. Without the solid additive the reaction mixture changed from powdery to a hard gum‐like mass with increasing conversion. This study highlights the potential of additive‐assisted milling as a practical depolymerization strategy for large‐scale PET waste streams (bottles, textiles, etc.).

**FIGURE 19:**
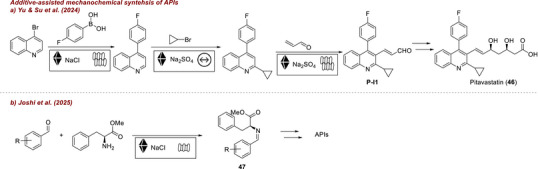
Additive‐assisted mechanochemical synthesis of APIs. (a) Mechanochemical synthesis of pitavastatin with NaCl and Na_2_SO_4_ as additives. (b) Mechanochemical synthesis of aldimines using SSE and NaCl as additive.

Another field where additive‐assisted mechanochemistry may exert major future impact is the synthesis of active pharmaceutical ingredients (APIs). Yu and Su [72] reported the full mechano‐synthetic sequence of a pitavastatin intermediate **PI‐1** using three additive‐assisted steps, demonstrating both scalability and a reduced environmental impact (Figure 19a). The Suzuki–Miyaura step was enabled by NaCl, while Na_2_SO_4_ facilitated both the Minisci

C–H alkylation and the oxidative Heck reaction. In a related contribution, Joshi et al. [73] developed a one‐pot, solvent‐free synthesis of aldimines **47** using single‐screw extrusion (SSE) (Figure 19b). This approach provides high‐value API precursors in yields up to >99% and showcases SSE as a scalable, energy‐efficient alternative to traditional batch imine synthesis. During optimization, the authors encountered mixing and solidification problems, common drawbacks in extrusion, which were successfully circumvented by solid additives such as NaCl. Together, these studies illustrate how additives are indispensable in industrial mechanochemistry, especially for enabling continuous manufacturing. Beyond synthesis, additive‐assisted mechanochemistry can also support (late‐stage) modifications [74] and even the prediction of degradation profiles [75] of APIs. They often help in countering lump formation and insufficient mixing among other limitations.

### 1.6 Non‐ionic Additives

#### 1.6.1 Silica (SiO_2_) as Main Additive

Silica (SiO_2_), in its different forms (silica gel, sand, quartz, etc.), has emerged as one of the most extensively investigated grinding auxiliaries. Its framework bears surface hydroxy groups, imparting a weakly acidic character and hydrogen‐bonding capability, which can explain its active role in some reactions, while in others it primarily acts as an adsorbent and inert filler.

**FIGURE 20:**
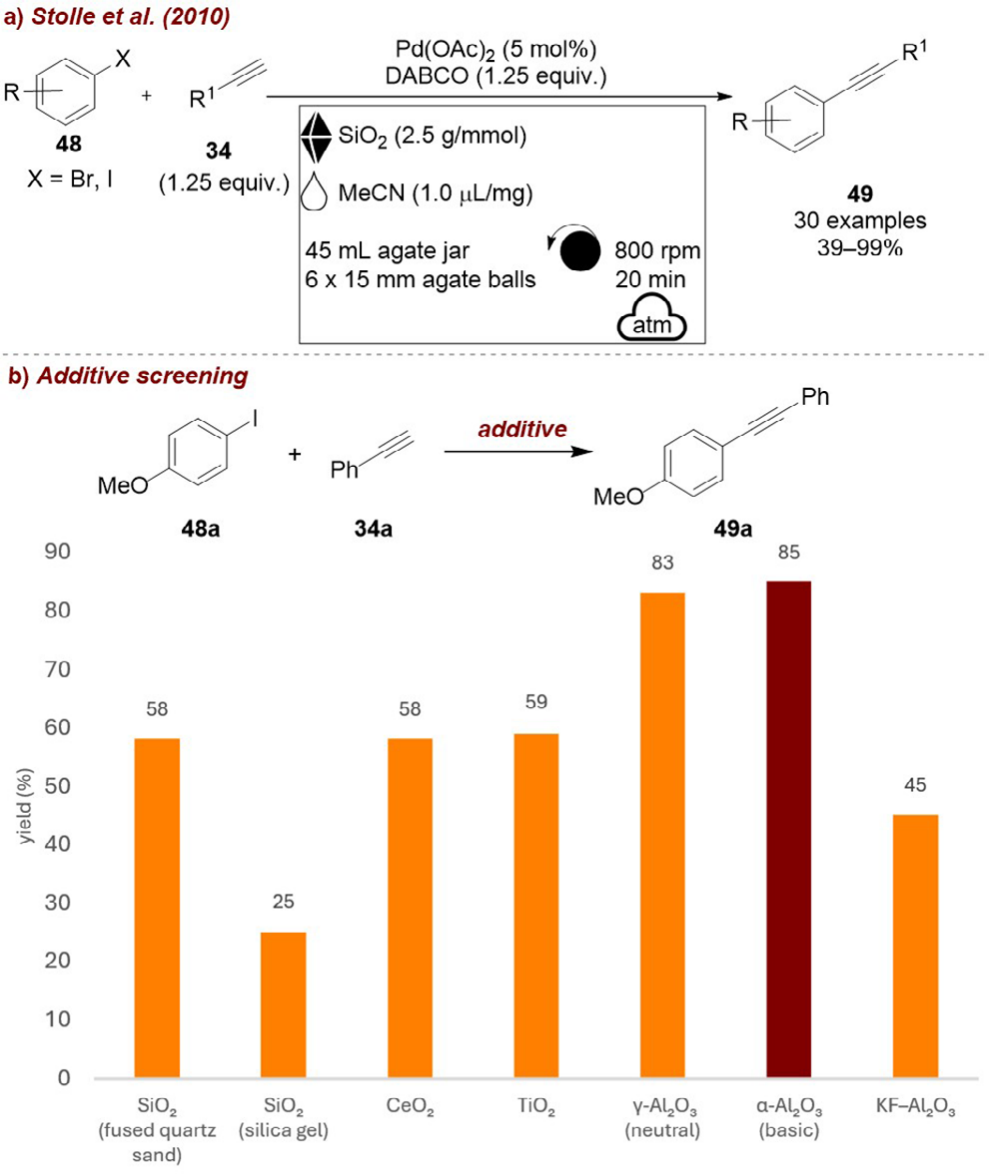
(a) Mechanochemical copper‐, ligand‐, and solvent‐free Sonogashira coupling reaction in a planetary mill. (b) Additive screening. Reaction conditions: **48a** (2.0 mmol), **34a** (1.25 mmol), DABCO (1.25 equiv.), Pd(OAc)_2_ (5 mol%), *additive* (2.5 g/mmol), MeCN (𝜂 = 0.24 µL/mg), agate jar (45 mL), agate balls (6 × 15 mm), planetary mill: 800 rpm, 20 min. Yield determined by GC‐FID analysis.

In 2010, Stolle and co‐workers [76] reported one of the earliest examples for the use of covalent milling auxiliaries in mechanochemical reactions, developing a copper‐, ligand‐, and solvent‐free Sonogashira coupling protocol in a planetary mill. The catalytic system, based on palladium with DABCO acting as both base and co‐catalyst, enabled the coupling between aryl iodides or bromides **48** and alkynes **34** yielding the corresponding product **49** (Figure 20a). In this case, fused quartz sand was introduced as an inert milling additive, also referred to as an “auxiliary grinding agent.” Although the study was primarily conducted using silica as grinding auxiliary, a subsequent investigation on the influence of different additives revealed that alumina (both neutral and basic) provided notably better results compared to other tested oxides. In fact, the use of alumina proved essential for extending the protocol to aryl bromides, for which silica was no longer effective.

**FIGURE 21:**
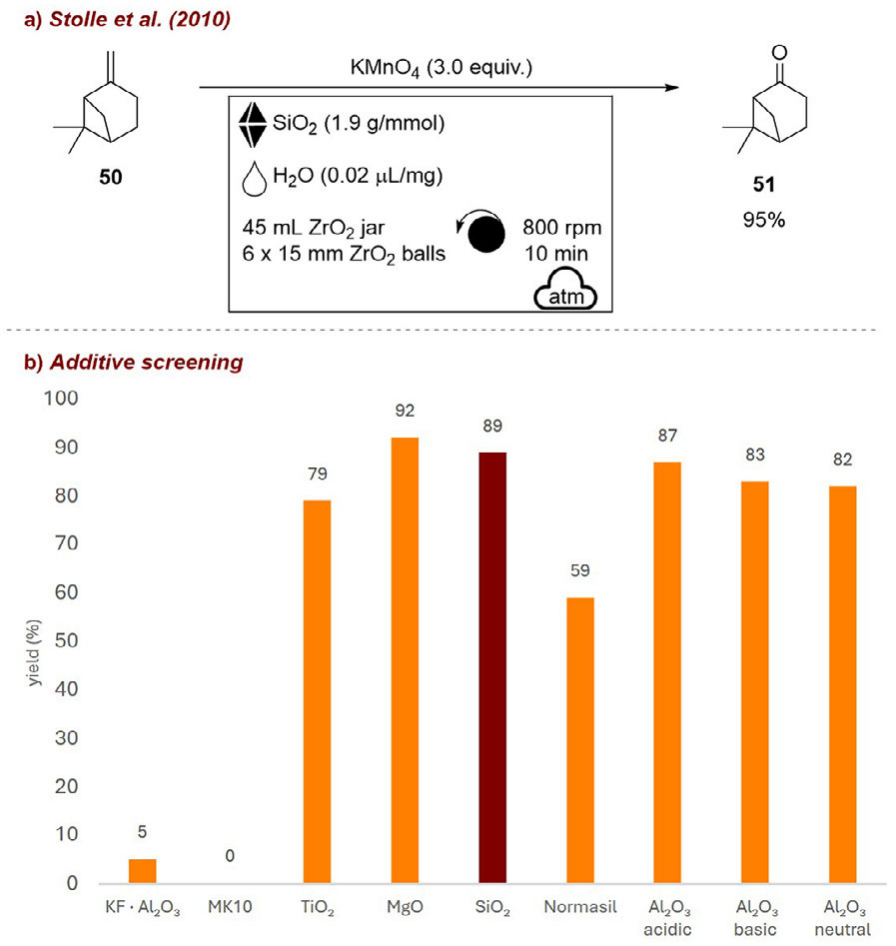
(a) Mechanochemical selective oxidation of β‐pinene **50** to nopinone **51** with KMnO_4_. (b) Additive screening. Reaction conditions: **50** (2.0 mmol), KMnO_4_ (3 equiv.), *additive* (1.9 g/mmol), H_2_O (𝜂  =  0.02 µL/mg), ZrO_2_ jar (45 mL), ZrO_2_ balls (6 × 15 mm), planetary mill: 800 rpm, 10 min. Yield determined by GC‐FID analysis.

Building on this concept, the same authors carried out a systematic study to clarify the role of inert milling auxiliaries, using the selective oxidation of β‐pinene **50** (Figure 21a) to nopinone **51** with KMnO_4_ as a model transformation [77]. Because the process predominantly involved a liquid substrate, neat milling was considered inefficient in terms of energy transfer. To overcome this limitation, a range of oxides and silica materials were screened (Figure 21b), which the authors also described as “solid solvents.” Apart from MK10 and KF/Al_2_O_3_, most auxiliaries enabled high yields and selectivity under solvent‐free conditions, and in the end, silica was chosen as the main additive for its cost‐efficiency. By contrast, in the absence of any auxiliary, the reaction did not proceed, underscoring the crucial role of the additive in enabling effective energy transfer.

**FIGURE 22:**
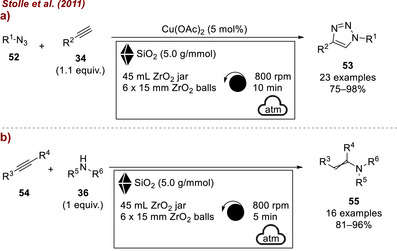
(a) Copper‐catalyzed click reactions in planetary mill assisted by SiO_2_ additive. (b) Solvent‐free addition of amines **36** to activated alkynes assisted by SiO_2_ additive. Product **55** represents both *Z* and *E* products.

In 2011, the same group further demonstrated the utility of fused quartz sand as a milling auxiliary in mechanochemical transformations. The additive enabled efficient energy transfer and proper filling of small‐scale milling jars and was applied to both the Huisgen 1,3‐dipolar cycloaddition between alkynes **34** and azides **52** for the synthesis of triazoles **53** [78] (Figure 22a) and the synthesis of enamines **55** from activated alkynes **54** and amines **36** [79] (Figure 22b). In both cases, the quartz sand adsorbed the liquid reagents and ensured effective mixing, which was essential for achieving efficient reaction outcomes.

**FIGURE 23:**
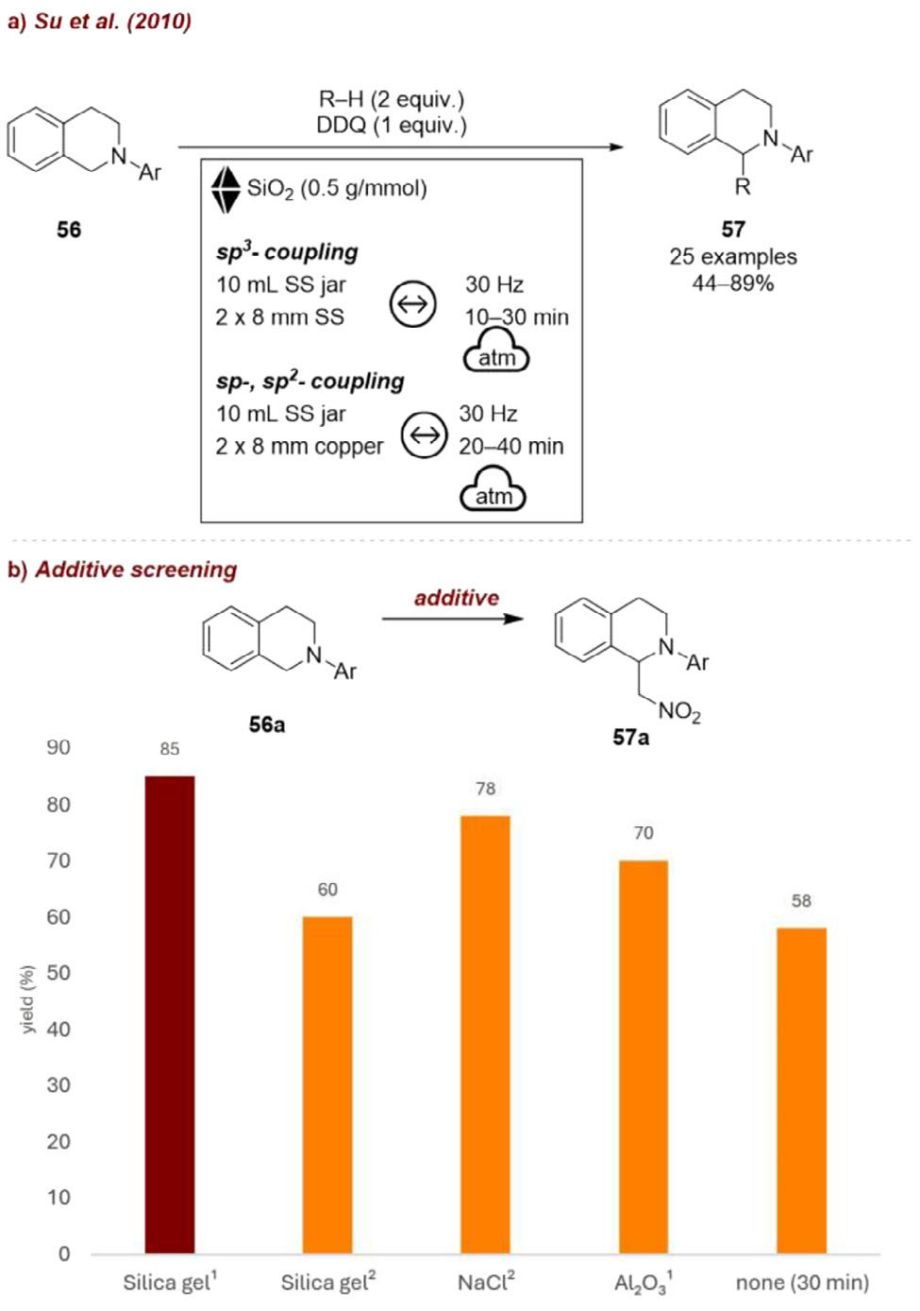
(a) Mechanochemical cross‐dehydrogenative coupling reactions under ball‐milling conditions with DDQ. (b) Additive screening. Reaction conditions: **56a** (1.0 mmol), nitromethane (2 equiv.), DDQ (1 equiv.), *additive* (^1^0.5 or^2^ 2.0 g/mmol), SS jar (10 mL), SS balls (2 × 8 mm), mixer mill: 30 Hz, 10 min. Isolated yield.

Another example for silica as a grinding auxiliary in mechanochemical reactions was provided by Su and co‐workers [80] (Figure 23a). First, they reported the use of additives as grinding auxiliaries in cross‐dehydrogenative coupling reactions under ball‐milling conditions with 2,3‐dichloro‐5,6‐dicyano‐1,4‐benzoquinone (DDQ). The authors demonstrated the effectiveness of milling auxiliaries in the coupling of tetrahydroisoquinolines **56** with sp^3^‐, sp^2^‐, and sp‐hybridized fragments. Notably, the coupling with sp^3^ fragments proceeded efficiently even without any metal catalyst, whereas reactions with sp^2^ and sp fragments required copper balls, which acted in direct mechanocatalysis. Silica, NaCl, and alumina were tested as milling auxiliaries, all of which significantly improved reaction efficiency, raising the yield from 58% in 30 min under neat conditions to over 70% in just 10 min (Figure 23b). Among these, silica (0.5 g/mmol) was the most effective, providing 85% of product **57a** in 10 min. The authors proposed that the auxiliary serves both as a grinding aid and as an adsorbent for the reagents. However, excessive amounts of additive (up to 2 g/mmol) led to a rapid decrease in yield due to reagent dilution. In later investigations the same group extended this methodology by employing silica as a grinding auxiliary and introducing a chiral ligand that interacted with the copper milling media, thereby rendering the sp^3–^sp coupling enantioselective [81].

**FIGURE 24:**
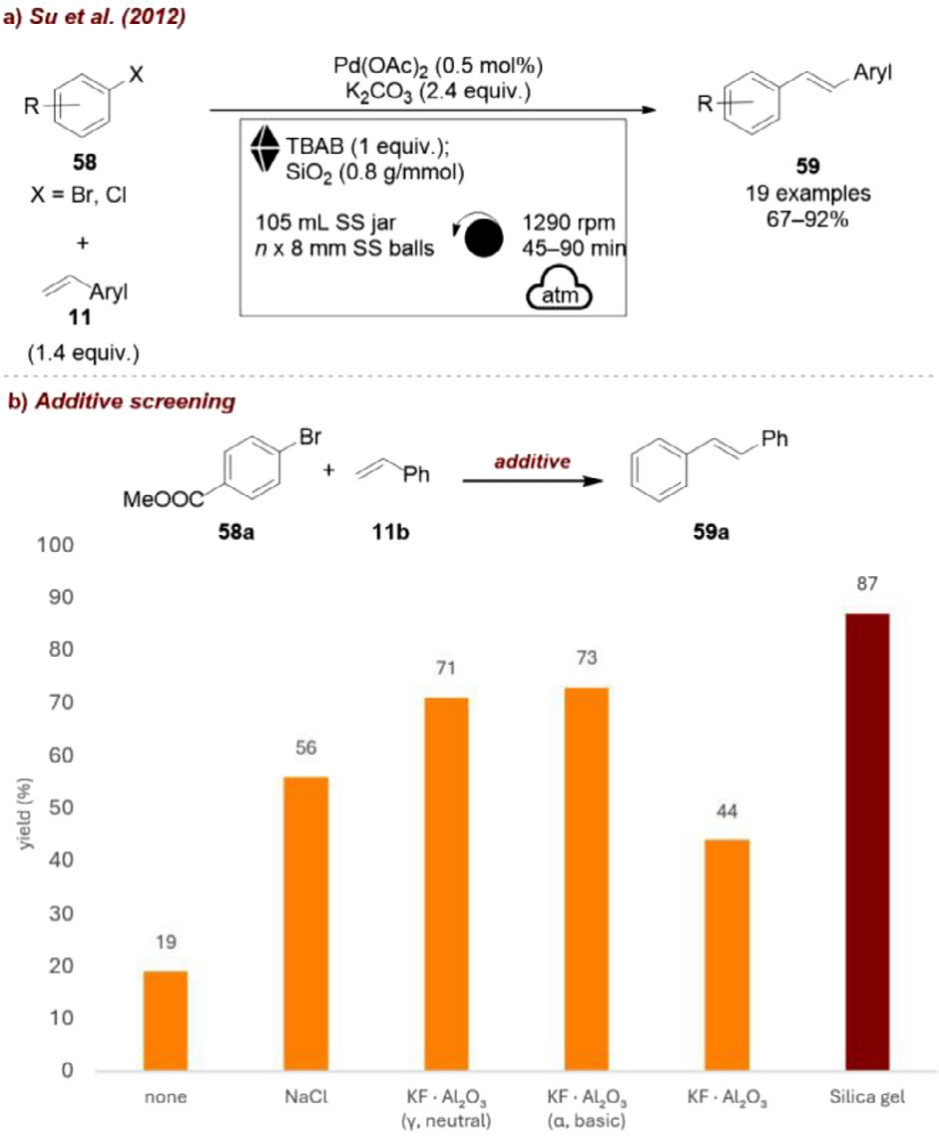
(a) Mechanochemical Pd‐mediated synthesis of *(E)‐*stilbene derivatives with silica as a grinding auxiliary. (b) Additive screening. Reaction conditions: **58a** (5.0 mmol), **11b** (1.4 equiv.), Pd(OAc)_2_ (0.5 mol%), K_2_CO_3_ (2.4 equiv.), TBAB (1 equiv.), *additive* (0.8 g/mmol), SS jar (105 mL), SS balls (8 mm, number unspecified), planetary mill: 1290 rpm, 45 min. Isolated yields.

In another instance, they reported the palladium‐mediated synthesis of *(E)‐*stilbene derivatives by solvent‐free ball milling, in which silica was again employed as a grinding auxiliary [82]. The coupling reaction was conducted in a planetary mill and was carried out between aryl halides **58** and styrenes **11** using Pd(OAc)_2_ as the catalyst to afford the corresponding stilbenes **59** (Figure 24a). In this study, two types of additives were used: (1) tetrabutylammonium bromide (TBAB), a crucial additive, as the reaction shows only negligible conversion in its absence, and (2) an inert additive serving as an adsorbent. According to the authors, the first additive stabilized the metal colloids formed in situ, preventing their aggregation into larger particles that are typically less active or inactive, while the second was required to ensure efficient mixing of the adsorbed materials. Among the auxiliaries tested, silica afforded the best results (85% yield, Figure 24b), although comparable outcomes were achieved with alumina (around 70%, both basic and acidic). By contrast, an ionic salt like NaCl gave lower efficiency (56%), yet still improved the performance compared to neat grinding, which only afforded a 19% yield.

**FIGURE 25:**
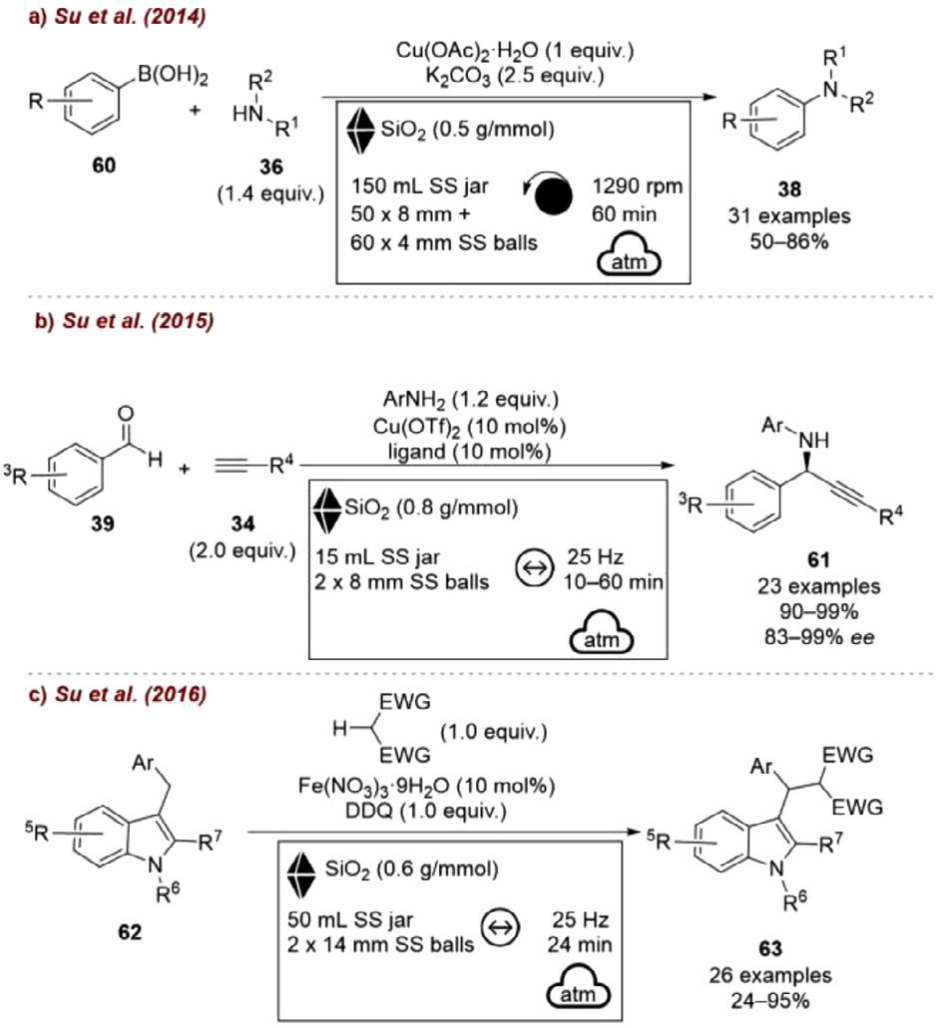
Other representative examples of SiO_2_ used as a grinding auxiliary: (a) Copper‐catalyzed C–N cross‐coupling between boronic acids **60** and amines **36**. (b) Copper‐catalyzed enantioselective A^3^ coupling. (c) Iron‐catalyzed cross‐dehydrogenative coupling of 3‐benzylic indoles **62**.

In follow‐up studies, Su and co‐workers further established the beneficial role of silica across a range of mechanochemically catalyzed transformations, including the copper‐catalyzed C–N cross‐coupling of boronic acids **60** with amines **36** [83] (Figure 25a), the Cu‐catalyzed enantioselective aldehyde‐alkyne‐amine reaction (A^3^ coupling) [84] (Figure 25b), the iron‐catalyzed cross‐dehydrogenative coupling of 3‐benzylic indoles **62** [85] (Figure 25c), and others [86, 87]. Altogether, these results demonstrated in a compelling way that silica, when employed as a grinding auxiliary, can decisively improve the performance of numerous mechanochemical transformations.

**FIGURE 26:**
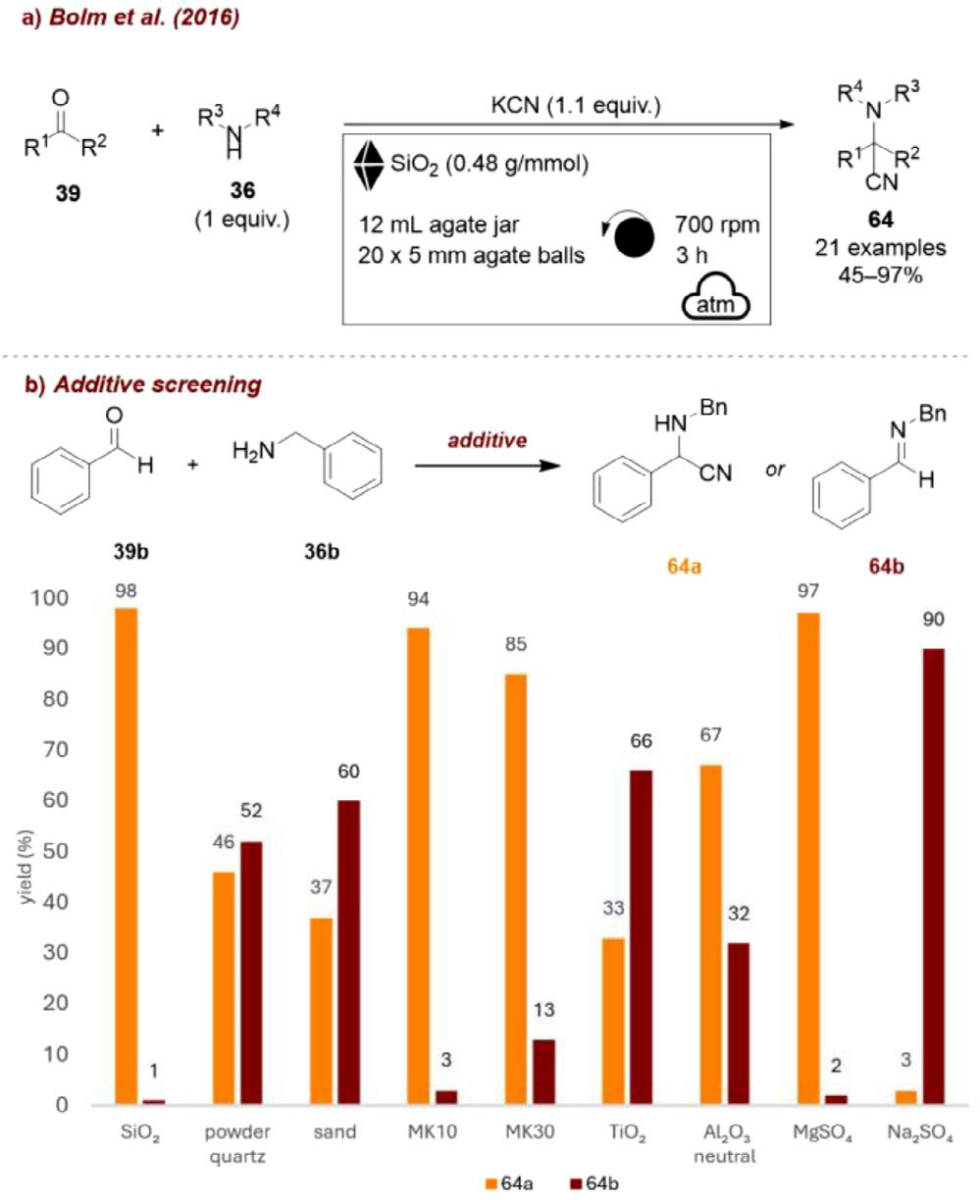
(a) Mechanochemical multicomponent Strecker reaction with SiO_2_ as additive. (b) Additive screening. Reaction conditions: **39b** (0.5 mmol), **36b** (1 equiv.), KCN (1.1 equiv.), *additive* (0.48 g/mmol), agate jar (12 mL), agate balls (20 × 5 mm), planetary mill: 700 rpm, 3 h. Yield determined by ^1^H NMR spectroscopy.

Subsequently, Bolm and co‐workers highlighted other distinct effects of silica additive in mechanochemical reactions, disclosing its remarkable influence on the multicomponent Strecker reaction. In the first work [88], the synthesis of α‐aminonitriles was shown to be facilitated by the presence of silica, which acted not only as a grinding auxiliary but also as a Lewis acid and moisture adsorbent within the milling mixture. The additive effect was examined using benzaldehyde **39b** and benzylamine **36b** in the presence of potassium cyanide (KCN, Figure 26a). Strikingly, while silica gel provided complete selectivity toward the final product **64a** (Figure 26b) the use of less Lewis‐acidic additives such as quartz powder, sand, or Na_2_SO_4_ resulted in significant amounts of the imine intermediate **64b** (52, 60, and 91%, respectively), suggesting that the additive's acidity plays an important role in the efficiency of the cyanation step. Other Lewis‐acidic additives, such as MK10 or MgSO_4_, also afforded **64a** with full selectivity. Interestingly, the authors further demonstrated the recyclability of the additive, showing that it remained active over two subsequent milling cycles after simple filtration, washing, and drying.

**FIGURE 27:**
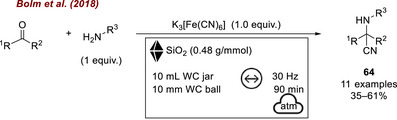
Mechanochemical activation of iron cyano complexes for the synthesis of α‐amino acid derivatives through in situ HCN generation, with SiO_2_ as a key additive.

In a second study, Bolm's group [89] extended the mechanochemical Strecker synthesis to possible prebiotic scenarios that could have contributed to the origin of life. In this study, prebiotic cyanide sources (such as cyanoferrate complexes and Prussian blue) were considered, which may have been activated by the physical impact of extraterrestrial bodies on early Earth. As in the previous case, silica—plausibly a material available in prebiotic environments—was employed as an additive and once again played a key role in directing the reaction selectivity toward the desired α‐aminonitrile (Figure 27). The authors demonstrated that during the milling of K_3_[Fe(CN)_6_], gaseous hydrogen cyanide (HCN) can be released, and that this release is specifically triggered by the addition of silica gel, owing to its intrinsic acidity. In this context, the additive not only guided selectivity toward the target product but also acted as an activator for the in situ generation of reactive HCN directly from a prebiotic cyanide source. This work exemplifies how mechanochemical activation can mimic geochemical processes relevant to the origin of life, highlighting the chemical functionality of common minerals such as silica beyond their traditional mechanical role.

**FIGURE 28:**
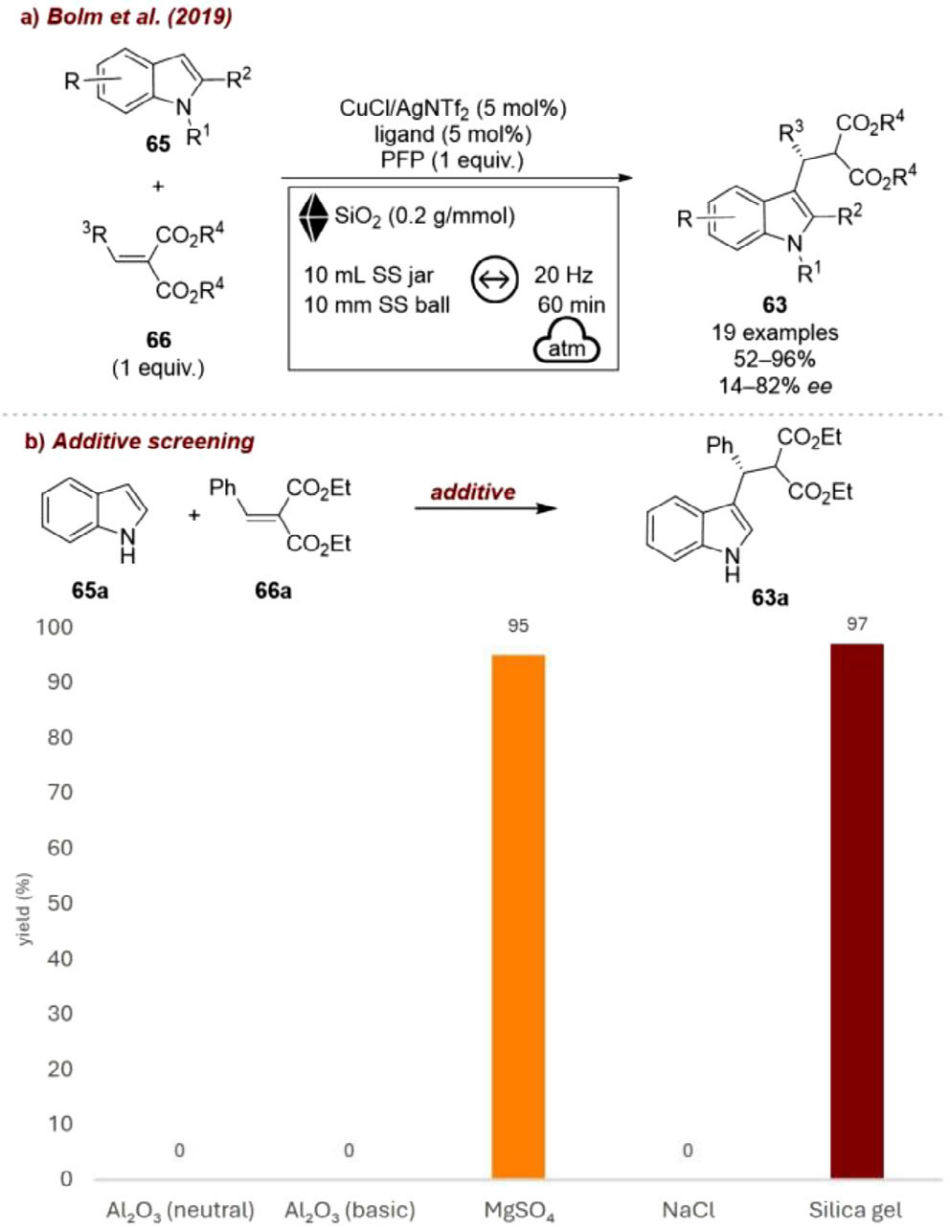
(a)Mechanochemical copper‐catalyzed asymmetric Michael‐type Friedel–Crafts alkylation of indoles with SiO_2_ as grinding auxiliary. (b) Additive screening. Reaction conditions: **65a** (0.3 mmol), **66a** (1 equiv.), CuCl/AgNTf_2_ (5 mol%), ligand (5 mol%), *additive* (0.2 g/mmol), SS jar (10 mL), SS ball (10 mm), mixer mill: 20 Hz, 60 min. Yield determined by ^1^H NMR spectroscopy.

In 2019, Bolm and co‐workers [90] reported the copper‐catalyzed asymmetric Michael‐type Friedel–Crafts alkylation of indoles with arylidene malonates. In this study, a Cu(I)–bis(oxazoline) (BOX) complex catalyzed the enantioselective alkylation of indoles **65** with benzylidene malonates **66** to afford the corresponding products **63** under ball‐milling conditions (Figure 28a). Adding pentafluorophenol (PFP) the enantioselectivity of the reaction was further improved. Silica was employed as a grinding auxiliary, likely due to the presence of liquid reagents in the mixture. Notably, however, the reaction proceeded with comparable yields and enantiomeric excesses even in the absence of an additive. When other auxiliaries were tested, MgSO_4_ provided results similar to silica, whereas alumina (both basic and neutral) and NaCl completely suppressed the transformation.

**FIGURE 29:**
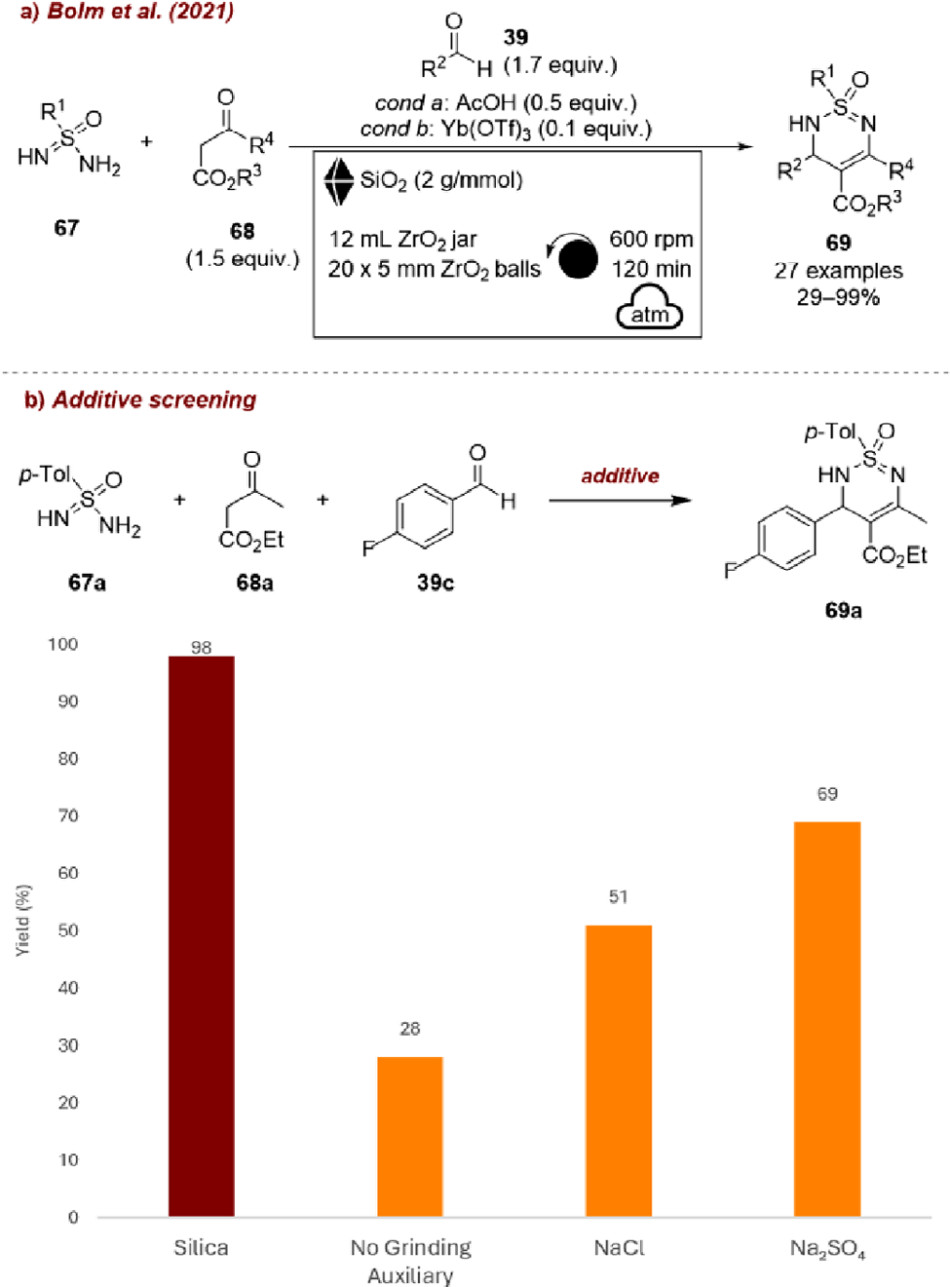
(a) Mechanochemical protocol for the synthesis of cyclic sulfonimidamide derivatives through a Biginelli‐type reaction with SiO_2_ as grinding auxiliary. (b) Additive screening. Reaction conditions: **67a** (0.10 mmol), **39c** (1.7 equiv.), **68a** (1.5 equiv.), AcOH (0.5 equiv.), *additive* (2 g/mmol), ZrO_2_ jar (12 mL), ZrO_2_ balls (20×5 mm), planetary mill: 600 rpm, 60 min. Yield determined by quantitative ^19^F NMR spectroscopy.

The same group [91] also reported a mechanochemical protocol for the synthesis of cyclic sulfonimidamide derivatives through a Biginelli‐type multicomponent reaction between sulfonimidamide **67a**, aldehyde **39c**, and β‐keto ester **68a**, affording the corresponding product **69a** (Figure 29a). In this case, the optimal outcome was achieved by combining acetic acid with silica as grinding auxiliary (Figure 29b). Other solid additives such as Na_2_SO_4_ or NaCl proved detrimental. The authors highlighted the mild acidity of silica as crucial to the reaction, along with its complementary roles as a desiccant and grinding auxiliary.

**FIGURE 30:**
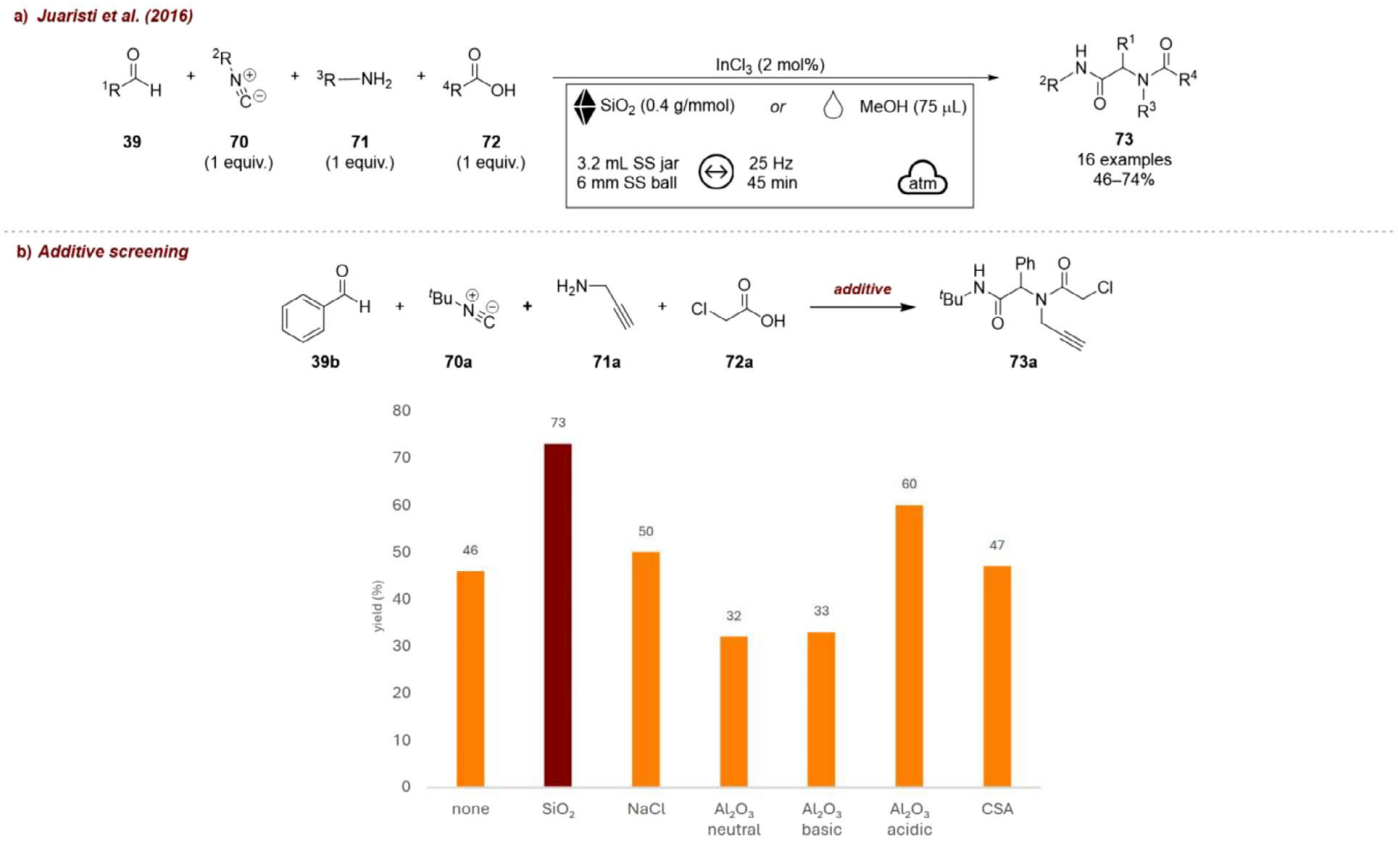
(a) Mechanochemical synthesis of Ugi (four‐component reaction) adducts. (b) Additive screening. Reaction conditions: **39b** (0.50 mmol), **70a** (1 equiv.), **71a** (1 equiv.), **72a** (1 equiv.), InCl_3_ (2 mol%), *additive* (0.4 g/mmol), SS jar (3.2 mL), SS ball (6 mm), mixer mill: 25 Hz, 90 min. Isolated yield. CSA = cellulose‐chlorosulfonic acid.

In another example, Juaristi and Polindara‐García [92] reported the multicomponent mechanochemical synthesis of Ugi (four‐component reaction) and Passerini (three‐component reaction) adducts. Among the two, only the Ugi reaction involved the use of additives (Figure 30a). Specifically, isocyanide **70a**, benzaldehyde **39a**, chloroacetic acid **72a**, and propargylamine **71** were combined under indium(III) chloride catalysis to afford the corresponding Ugi product **73a** (Figure 30b). During optimization, the effect of various additives (e.g., NaCl, Al_2_O_3_, etc. Figure 30b) was systematically investigated. Interestingly, silica emerged as the most effective additive, improving the yield of **73a** from 46% to 73%. The authors proposed that this beneficial effect arises from the weak Brønsted acid nature of silica: the hydroxy groups of the silica framework may activate the formation of the initial imine intermediate through hydrogen bonding, thereby facilitating the subsequent assembly of the Ugi adduct. Nevertheless, despite the positive role of silica, the authors ultimately favored an LAG protocol, which delivered comparable yields (74%) while avoiding the additional work‐up step required when using a solid additive.

**FIGURE 31:**
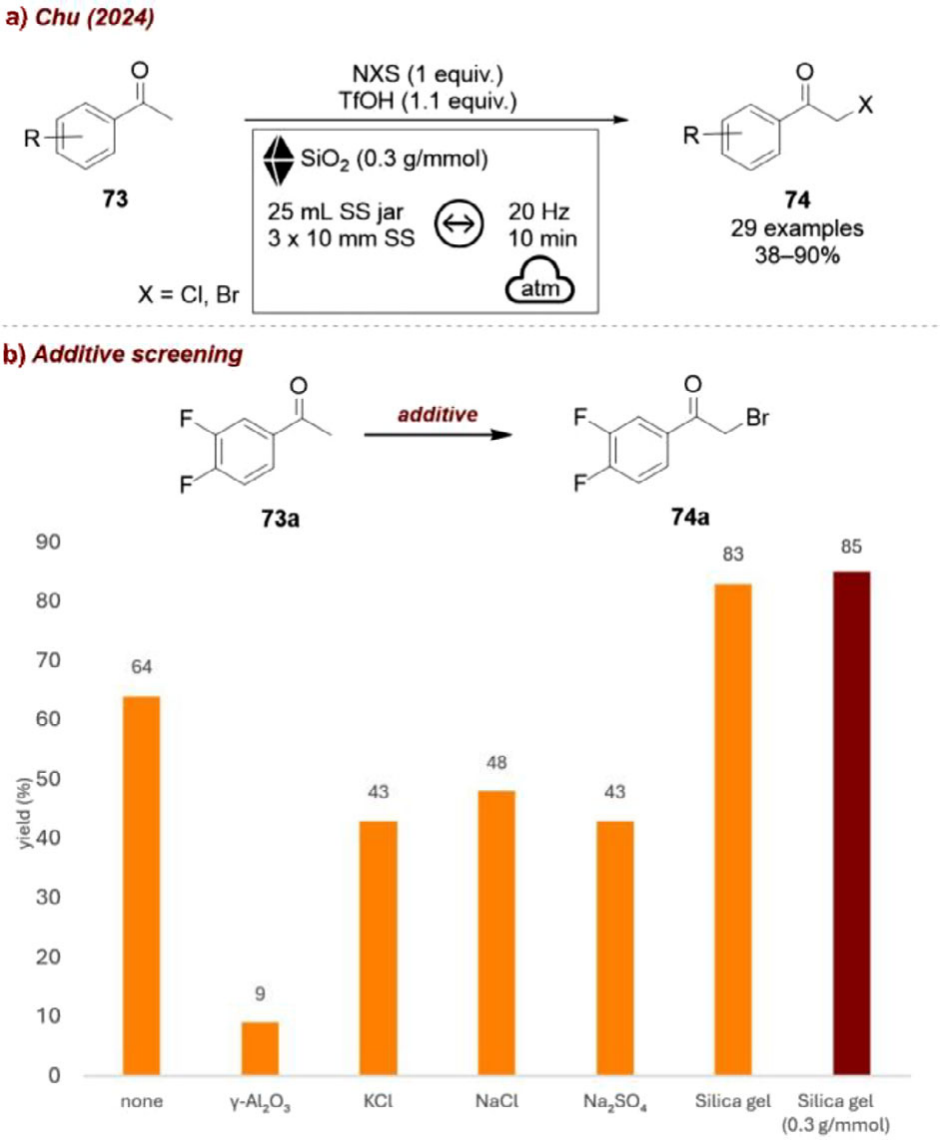
(a) Mechanochemical method for α‐halogenation of ketones. (b) Additive screening. Reaction conditions: **73a** (0.50 mmol), NBS (1 equiv.), TfOH (1.1 equiv), additive (0.4 g/mmol), SS jar (25 mL), SS balls (3×10 mm), mixer mill: 30 Hz, 30 min. Yield determined by HPLC analysis.

Another case was reported by Chu and co‐workers, who achieved an efficient mechanochemical method for α‐halogenation of **73**, (Figure 31a). The work could be extended by a biocatalysis step to obtain chiral aromatic epoxides from the corresponding ketones [93]. A series of inert additives were screened, including γ‐Al_2_O_3_, KCl, NaCl, and Na_2_SO_4_, all of which proved detrimental, decreasing the yield (8%–48%) compared to the additive‐free reaction (64%). By contrast, silica as an additive markedly improved the outcome, boosting the yield of **74a** up to 84%. Careful selection of the milling parameters and the grinding auxiliary ultimately enabled the process to reach 91% yield for the mechanochemical step, delivering the intermediate for the subsequent biocatalytic epoxidation in only 10 min and with high efficiency.

**FIGURE 32:**
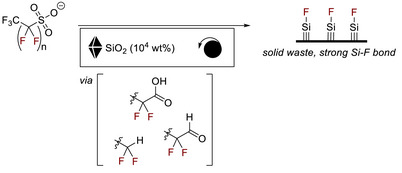
Environmental detoxification with additive‐assisted grinding: Mechanochemical destruction of PFSAs.

Another example underscoring the industrial‐scale environmental potential of additive‐assisted mechanochemistry is its use in destroying persistent toxic compounds. To contribute to environmental detoxification, Gobindlal and Sperry [94] showed that silica is highly effective for the mechanochemical destruction of perfluorosulfonic acids (PFSAs, Figure 32), a harmful subclass of polyfluoroalkyl substances (PFAS) linked to serious health risks. During milling, silica generates mechanoradicals and silanols that drive halide abstraction and C–C bond scission, ultimately forming stable Si–F bonds on the surface and preventing hazardous fluoride byproducts. This solvent‐free, ambient‐condition, additive enabled process yields benign solids and achieves near‐complete PFSA destruction, demonstrating an environmentally friendly and scalable strategy to moderate the long‐term impact of these “forever chemicals.”

### 1.7 Carbohydrate‐Based Additives

Another class of non‐ionic additives that has proven highly effective in various mechanochemical transformations is that based on carbohydrates. Carbohydrates are an abundant class of naturally occurring compounds characterized by high density of polar functional groups, extensive hydrogen‐bonding capability, and inherent chirality. These features make them particularly appealing as environmentally benign additives, capable of influencing reaction environments through both physical and supramolecular interactions.

In an early example where the additive does not directly influence the organic synthesis step, Hapiot and co‐workers [95] showed that cyclodextrins (CDs) are crucial milling additives in the mechanochemical preparation of gold NPs (AuNPs) and their application in nitroarene reduction. CDs stabilize the NPs, control aggregation, and enhance substrate–AuNP interactions through supramolecular complexes, leading to high selectivity and yields. The protocol remains effective even with in situ AuNP formation and shows good performance over multiple recycles, highlighting the dual role of CDs as a stabilizer and dispersing agent.

**FIGURE 33:**
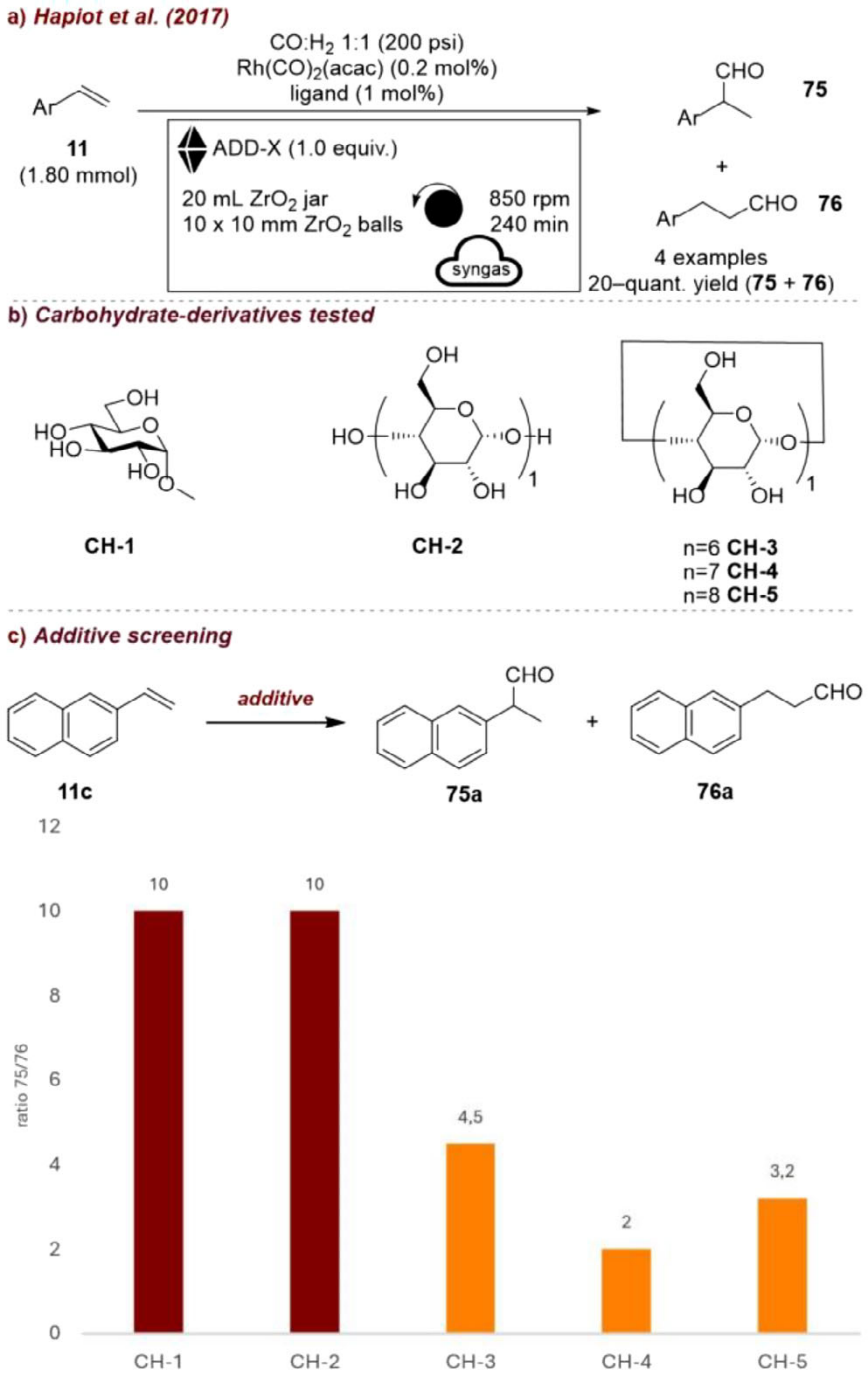
(a) Mechanochemical hydroformylation of alkenes in a planetary ball mill with CDs as grinding auxiliaries. (b) Additive screening. Reaction conditions: **11c** (1.8 mmol), Rh(CO)_2_(acac) (0.2 mol%), PPh_3_ (1 mol%), CO/H_2_ (1:1) (220 psi), *additive* (1 equiv.), ZrO_2_ jar (20 mL), ZrO_2_ balls (10 × 10 mm), planetary mill: 850 rpm, 4 h. Graphic reports the molar ratio of **75a** to **76a** measured under the indicated reaction conditions. Ratio calculated over yield determined by ^1^H NMR spectroscopy.

The same group later developed a mechanochemical protocol for the hydroformylation of alkenes [96] in a planetary ball mill. In this example, an alkene **11** was placed in a reaction vessel under a pressurized CO/H_2_ gas mixture, yielding the two regioisomeric products **75** and **76** (Figure 33a). Both cyclic (**CH‐1**, **CH‐2**, Figure 33b) and acyclic (**CH‐3–5**) saccharide additives were employed to facilitate the mechanochemical process (Figure 33c). The choice of additive proved crucial in directing the regioselectivity of the hydroformylation reaction, with acyclic saccharides favoring product **75** and cyclic saccharides, such as CDs, promoting product **76**, as shown in the graph, where the molar ratio of **75** to **76** decreases in favor of higher formation of **76**. The authors propose that this effect arises from the ability of cyclic saccharides to act as transient second‐sphere ligands around the catalyst center, exerting noncovalent, structure‐directing effects that favor hydroformylation at the less‐hindered position of the vinyl group, thereby promoting product **76**. In contrast, acyclic saccharides primarily enhance substrate diffusion within the solid mixture, leading to high selectivity for the branched product **75**. Similar benefits on the selectivity of mechanochemical reactions upon the addition of CDs were later demonstrated in the synthesis of chiral δ‐hydroxysulfones [97], iron‐catalyzed olefin oxidation [98], and aerobic oxidative Heck coupling [99].

**FIGURE 34:**
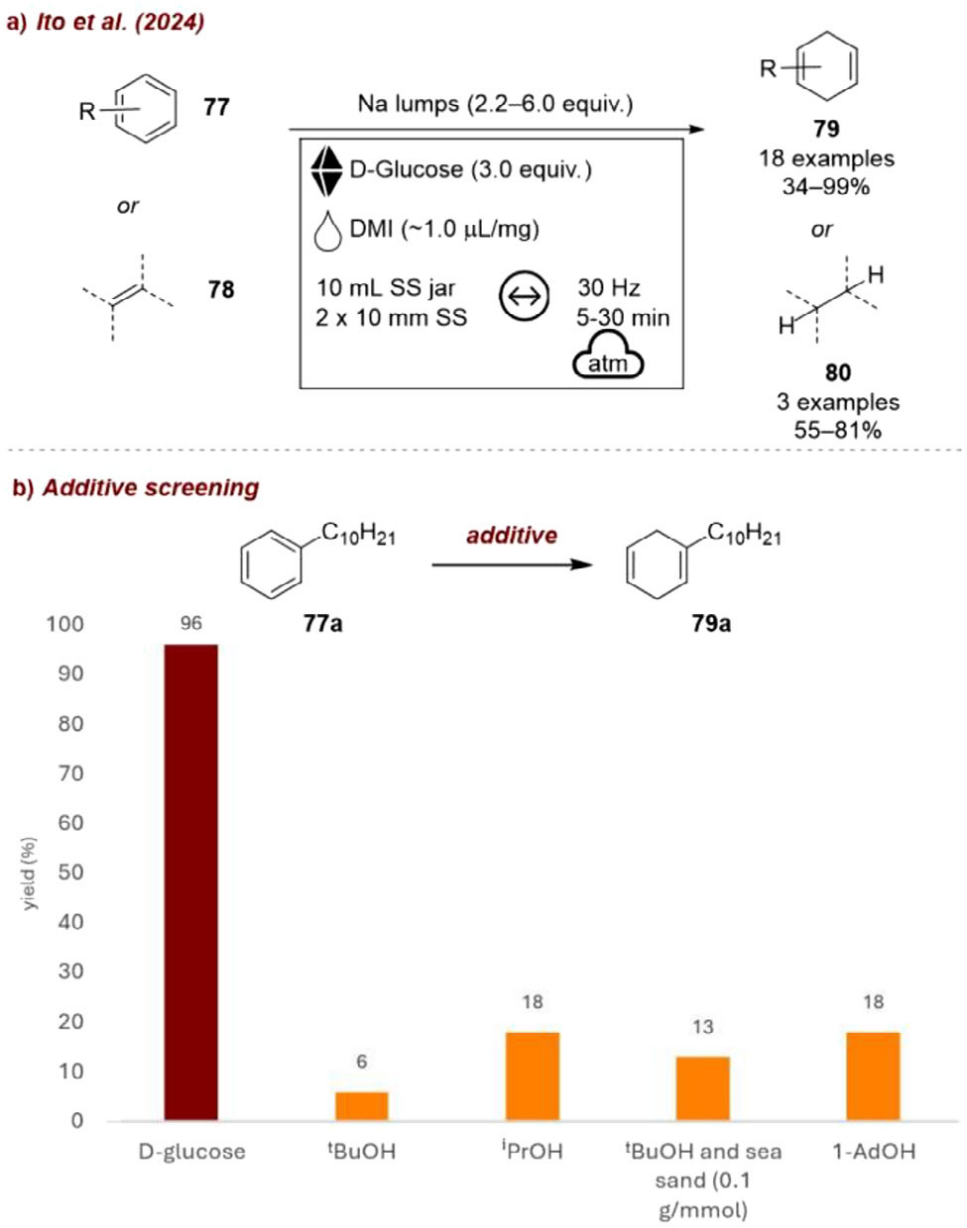
(a) Mechanochemical Birch‐type reduction of aromatic compounds employing D‐glucose as a proton source. (b) Additive screening. Reaction conditions: **77a** (1.0 mmol), Na lumps (6.0 equiv.), DMI (6.0 equiv.), *additive* (3 equiv.), SS jar (10 mL), SS balls (2 × 10 mm), mixer mill: 30 Hz, 15 min. Yield determined by ^1^H NMR spectroscopy.

A different use of carbohydrate‐based additives was reported by Kubota, Ito, and co‐workers [100], who recently developed a mechanochemical reduction of aromatic compounds employing d‐glucose as a proton source (Figure 34a). In this case, the additive played an active role in the reaction mechanism, acting as the proton donor. At the same time, it can also be considered a mechanochemical additive, since conventional alcohols typically used in Birch reductions (such as *^t^* BuOH, *^i^* PrOH, or even solid alcohols like 1‐adamantanol) failed to provide satisfactory yields of the desired product. According to the authors, efficient activation of sodium lumps required the reaction mixture to remain in the solid state, thereby ensuring the effective transfer of mechanical energy during milling. D‐Glucose, being only sparingly soluble in organic solvents, remained solid in the presence of 1,3‐dimethyl‐2‐imidazolidinone (DMI) and thus simultaneously functioned as both the proton source and a solidifying alcohol additive.

### 1.8 Other Non‐ionic Solids as Main Additives

**FIGURE 35:**
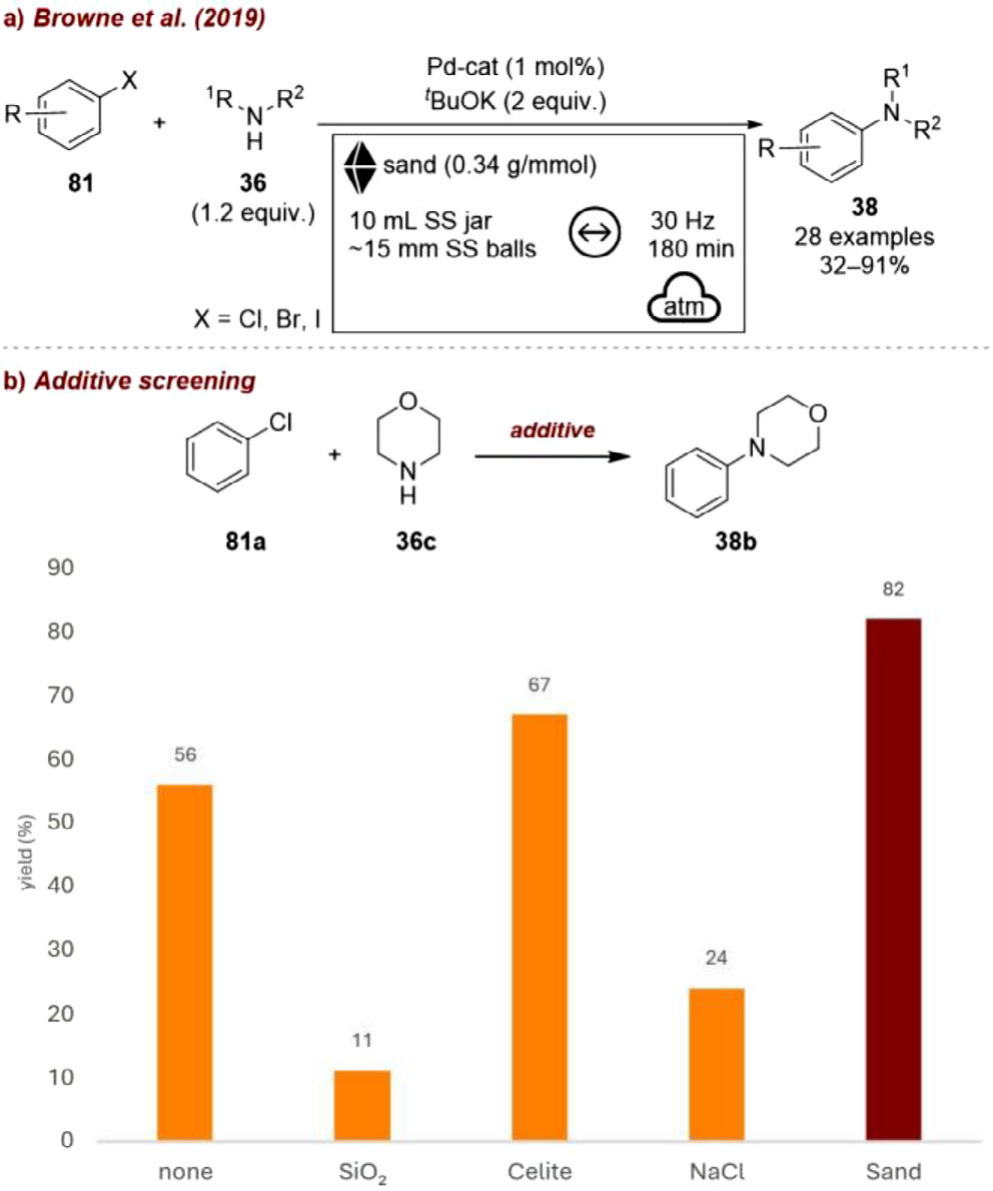
(a) Mechanochemical Buchwald–Hartwig reaction under aerobic conditions with sand as grinding auxiliary. (b) Additive screening. Reaction conditions: **81a** (1.0 mmol), **36c** (1.2 equiv.), Pd‐cat (2 mol%), *^t^* BuOK (2 equiv.), *additive* (300 wt%), SS jar (10 mL), SS ball (15 mm), mixer mill: 30 Hz, 3 h. Yield determined by ^1^H NMR spectroscopy.

In one instance, Browne and co‐workers reported a mechanochemical protocol for the Buchwald–Hartwig reaction, in which ball milling enabled the coupling to proceed efficiently under aerobic conditions [101]. In this study, aryl halides **81** were reacted with secondary amines **36** to afford the corresponding coupling products **38** (Figure 35a). In the absence of milling auxiliaries, the reaction delivered the product in 56% yield (Figure 35b). The addition of commonly used additives, such as silica gel or NaCl, instead resulted in a notable decrease in efficiency, with yields dropping below 24%. By contrast, the use of celite improved the outcome to 67%, and sand further enhanced the process to 82%, ultimately reaching up to 95% yield under optimized conditions. While the authors did not elaborate on the origin of this difference, one could hypothesize that the improved performance of sand compared to silica might be related to its lower surface acidity, which could otherwise interact with the secondary amine substrate. This example highlights how different forms of materials with similar chemical nature—but varying crystallinity, structure, and surface properties—can substantially influence the outcome of a mechanochemical reaction.

**FIGURE 36:**
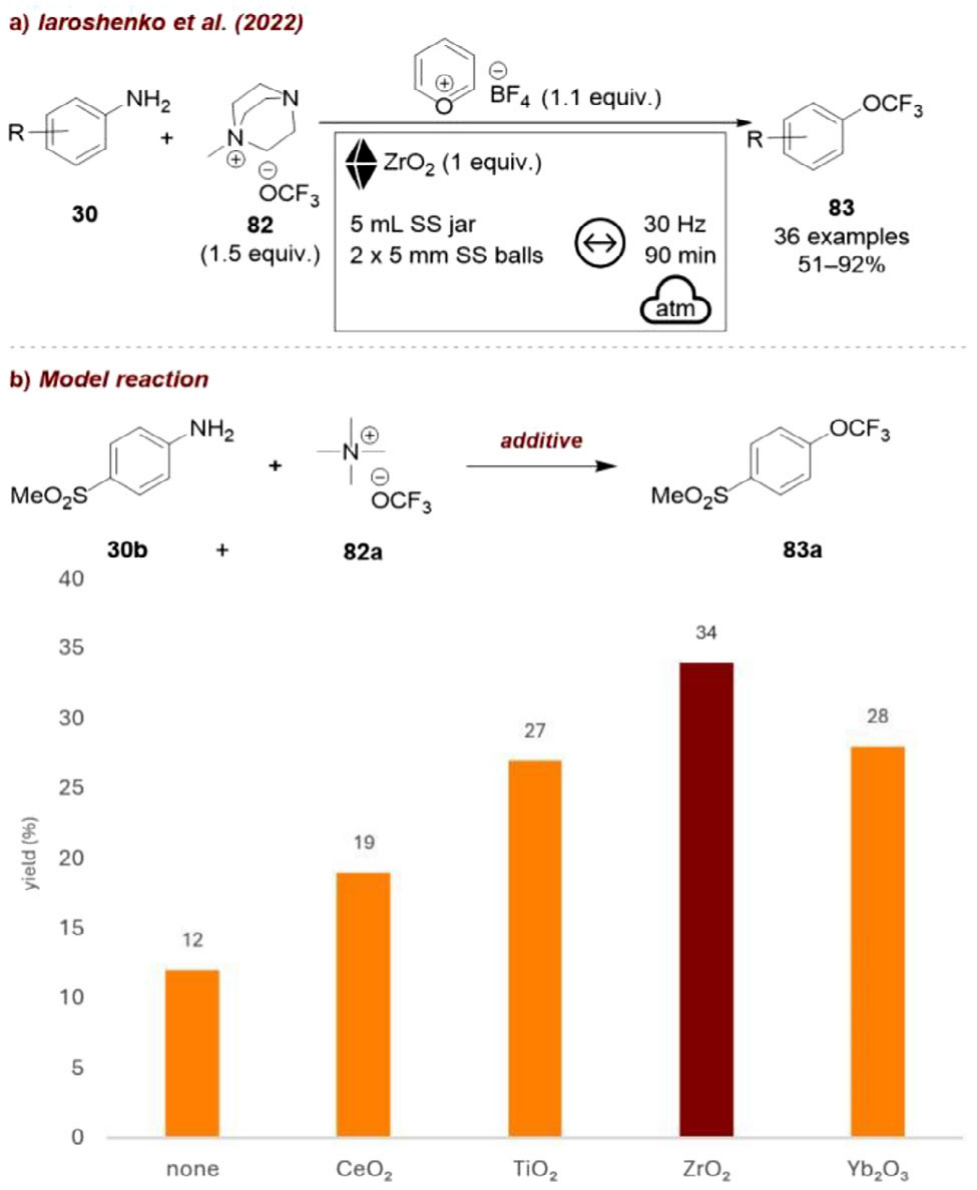
(a) Mechanochemical transformation of aromatic amines into aryl trifluoromethyl ethers with ZrO_2_ as grinding auxiliary. (b) Additive screening. Reaction conditions: **30b** (1.0 mmol), **Pyry BF_4_** (1.1 equiv.), **82a** (1.5 equiv.), *additive* (1 equiv.), SS jar (5 mL), SS balls (2 × 5 mm), mixer mill: 30 Hz, 1.5 h.

Other metal oxides have also been employed as additives in mechanochemical reactions. For example, Iaroshenko and co‐workers recently reported a mechanochemical protocol using pyrylium tetrafluoroborate (Pyry BF_4_) (Figure 36a) for the conversion of aromatic amines **30** into aryl trifluoromethyl ethers **83** [102] (Figure 36a). Since the starting materials were liquids and the neat reaction only afforded low yields (12%), the use of metal oxide additives was systematically investigated (Figure 36b). During this screening, the reaction between 4‐(methylsulfonyl)aniline **30b**, Pyry BF_4_ and tetramethylammonium trifluoromethylate **82a** as the OCF_3_ source was studied to afford the corresponding trifluoromethyl ether **83a**. Among those tested additives, only zirconia provided a substantial improvement, tripling the yield to 34%, and, in combination with optimization of the OCF_3_ anion source, ultimately delivering product **83a** in 78% yield. According to the authors, the superior performance of ZrO_2_ may be plausibly attributed to its stabilizing effect on the OCF_3_ anion, most likely through interactions of zirconium with both oxygen and fluorine atoms. Notably, the good affinity of four‐coordinate Zr to aliphatic C–F bonds could also contribute to this effect.

### 1.9 Piezoelectric Materials

Piezoelectric materials are noncentrosymmetric crystalline solids capable of converting external mechanical stimulation into electrical energy and vice versa. This significantly simplifies reaction set‐up for mechano‐redox reactions, as the materials act as an in situ source of electric potential, enabling redox reactions directly within the grinding jar [103]. Piezocatalysis is generally explained by two widely acknowledged models: the energy band theory and the screening charge effect. Although these mechanisms differ substantially, both emphasize the central role of the piezoelectric potential in facilitating chemical transformations. When piezoelectric materials are subjected to external mechanical forces or strain, the displacement of the cations and anions within the crystal lattice leads to a misalignment of positive and negative charge centers. This displacement generates a dipole moment—polarization. Upon alignment of these dipole moments in an orderly manner, a macroscopic internal electric field is established. Under continuous mechanical excitation, this field enables piezoelectric materials to drive chemical reactions [103, 104]. For details and discussions on the mechanisms, the reader is referred to specialized review articles [103, 104, 105]. To date, in redox mechanochemistry, barium titanate (BaTiO_3_), strontium titanate (SrTiO_3_), and piezoelectric MOFs are used. These materials can be activated by ultrasonication, ball milling, mechanical stirring, or even natural mechanical forces such as wind, tides, or water flow to drive redox reactions [104]. Besides the piezoelectric potential, another key property of these materials is grain size, which strongly affects the piezoelectric response and overall catalytic performance [106].

The piezoelectric effect has proven highly efficient in organic synthesis, bioapplications, water remediation, and small‐molecule catalysis [107, 108, 109]. In these systems, piezoelectric materials of various morphologies, ranging from powders to 2D films, are subjected to external mechanical excitation to trigger the desired chemical transformations [104]. Numerous reviews [110, 111, 112, 113] discuss organic mechanochemical reactions promoted by piezoelectric materials; therefore, this article highlights only the most representative examples to demonstrate the role and diversity of piezoelectric materials employed to date.

#### 1.9.1 Barium Titanate (BaTiO_3_)

**FIGURE 37:**
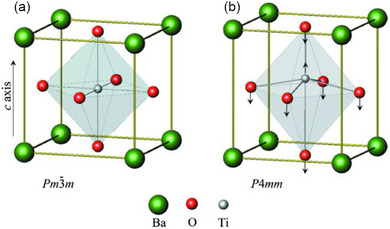
Crystal structure of (a) cubic and (b) tetragonal BaTiO_3_. The arrows in (b) denote the direction of displacements for Ti and O atoms with respect to the Ba atom. The material was reproduced from [121] with permission of the International Union of Crystallography.

Barium titanate (BaTiO_3_) exhibits four polymorphs depending on temperature: cubic (>120 °C), tetragonal (20–120 °C), orthorhombic (–90 to 5 °C), and rhombohedral (<‐90 °C, Figure 37). For mechanochemical applications, BaTiO_3_ is typically employed in the tetragonal crystalline phase [114, 115, 116], which is stable at room temperature for sufficiently large particles, typically >1 µm. The tetragonal distortion, and thus ferroelectricity, is gradually suppressed with decreasing particle size. Reported critical sizes for the disappearance of room‐temperature ferroelectricity of BaTiO_3_ range from 10 to 100 nm. Below this threshold BaTiO_3_ adopts an average cubic (nonpiezoelectric) structure at ambient conditions [117]. However, as seen below, some works still employ cubic BaTiO_3_ NPs (<100 nm), which raises an important question: how can cubic BaTiO_3_ NPs promote redox reactions under mechanochemical conditions, if they are nonpiezoelectric? This remains one of the central debates in the emerging field of mechanoredox chemistry. The ability of piezoelectric materials to trigger reactions under mechanical stress does not only depend on the polarizability of the material (dielectric constants d_33_) or its crystal structure. Parameters such as particle size of the piezomaterial should also be considered, since mechano‐induced electron transfer between piezoelectric materials and reactants are anticipated to occur at their interface.

As in other nanostructured ferroelectrics, the physical properties of BaTiO_3_ NPs differ markedly from those of bulk crystals, due to their high surface‐to‐volume ratio and enhanced structural flexibility [118]. BaTiO_3_ NPs below 100 nm can exhibit exceptionally high piezoelectric responses (large d_33_ values) under deformation. This enhancement is attributed to their ability to accommodate local distortions. In cubic NPs at room temperature, local Ti^4+^ displacements up to ∼0.15 Å have been observed [119] (Figure 37a), even though the average crystal structure appears cubic and centrosymmetric [117]. In bulk tetragonal BaTiO_3_ at room temperature, however, the off‐center displacement of Ti^4+^ within the oxygen octahedron is ∼0.05 Å (Figure 37b). This phenomenon, known as local symmetry breaking, gives rise to polar nanoregions (PNRs), nanoscale domains containing local dipoles caused by slight Ti^4+^ displacements. These PNRs enable piezoelectricity in macroscopically cubic BaTiO_3_ nanocrystals at room temperature and thus promote mechanoredox catalysis [117, 120].

**FIGURE 38:**
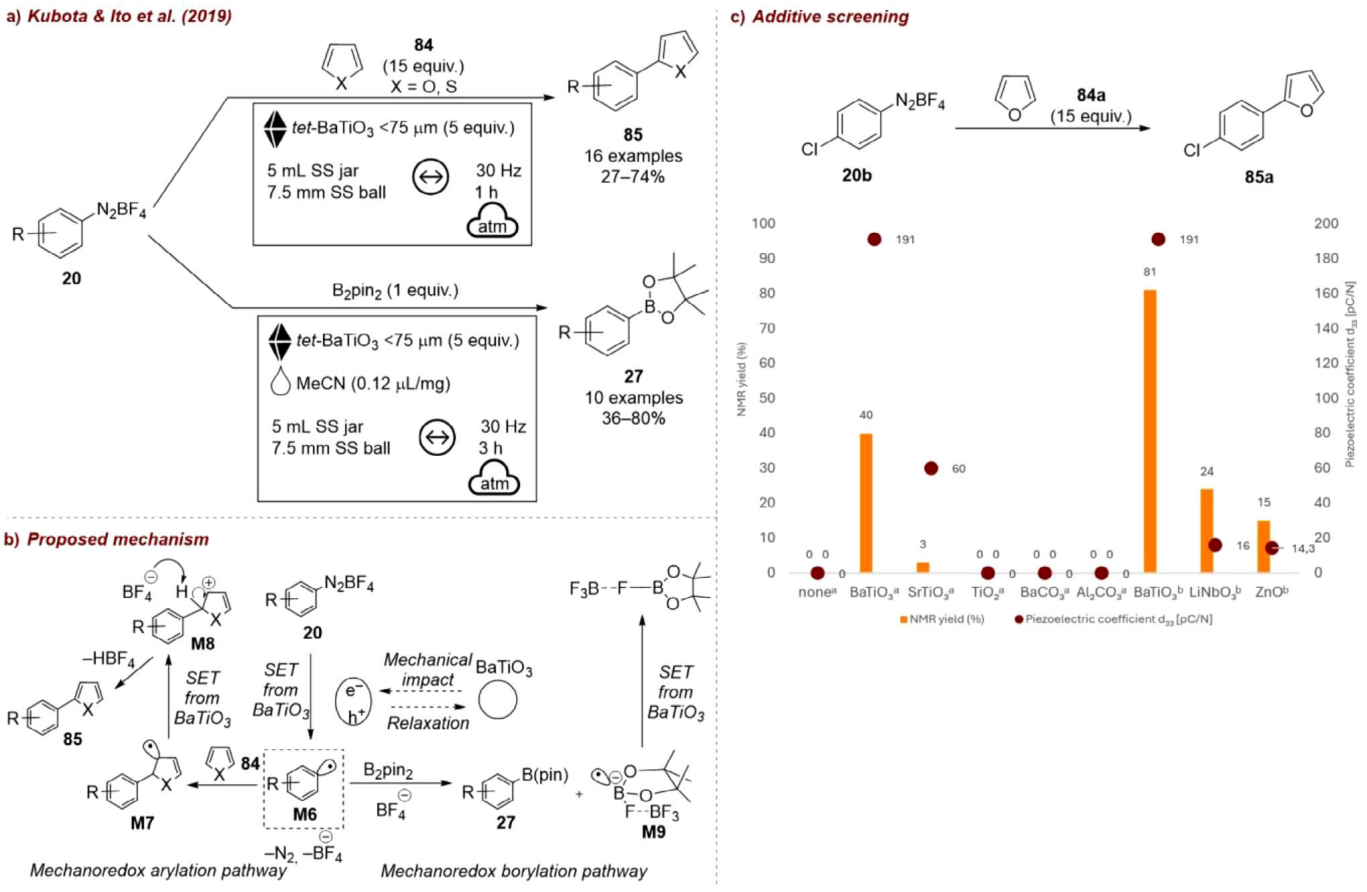
(a) Mechanochemical activation of aryl diazonium salts for heteroarene coupling and borylation reactions with *tet‐*BaTiO_3_. (b) Proposed mechanism. (c) Additive screening. Reaction conditions: **20b** (0.3 mmol), **84a** (15 equiv.), *additive* (5 equiv.), 1.5 mL SS jar, 5 mm SS ball, mixer mill: *frequency*, 1 h.

The first application of piezoelectric materials for organic synthesis under mechanochemical conditions was reported in 2019 by Kubota and Ito [122, 123], who applied BaTiO_3_ to activate aryl diazonium salts for heteroarene coupling and borylation reactions (Figure 38a). The group tested four piezoelectric materials: tetragonal BaTiO_3_, SrTiO_3_, LiNbO_3_, and ZnO and three nonpiezoelectric ceramics: BaCO_3_, Al_2_O_3_, and TiO_2_. No reaction occurred in the absence of BaTiO_3_, nor in the presence of the nonpiezoelectric materials (Figure 38c). In contrast, tetragonal BaTiO_3_ promoted the coupling reaction with 40% yield at 20 Hz, while SrTiO_3_, which exhibits piezoelectricity only under in‐plain strain, afforded only trace amounts of product. When the milling frequency was increased to 30 Hz, efficiency improved significantly: BaTiO_3_ gave 82% yield, while LiNbO_3_ and ZnO afforded lower yields of 24% and 15%, respectively. This is consistent with their comparatively weaker piezoelectric responses. The authors proposed a mechanism, analogous to photoredox catalysis (Figure 38b). The mechanical agitation of BaTiO_3_ generates a temporary electrochemical potential to reduce aryl diazonium salt **20** via a single‐electron transfer (SET), yielding aryl radical **M6**. Addition of **M6** to heteroarene **84** affords intermediate **M7**, which gets subsequently oxidized by the hole generated in agitated BaTiO_3_ to form carbocation intermediate **M8**. Following deprotonation, the arylation product **85** is obtained. In the borylation reaction, the generated radical **M6** reacts with bis(pinacolato)diboron (B_2_pin_2_) to form boryl substitution product **27** and radical intermediate **M9**. Upon oxidation of **M9** by the agitated BaTiO_3_, F–B(pin) is formed as a byproduct. While the catalyst could be recycled, a gradual reduction in substrate conversion was observed over time, despite minimal loss of mass, suggesting partial deactivation of the piezocatalyst during repeated milling.

**FIGURE 39:**
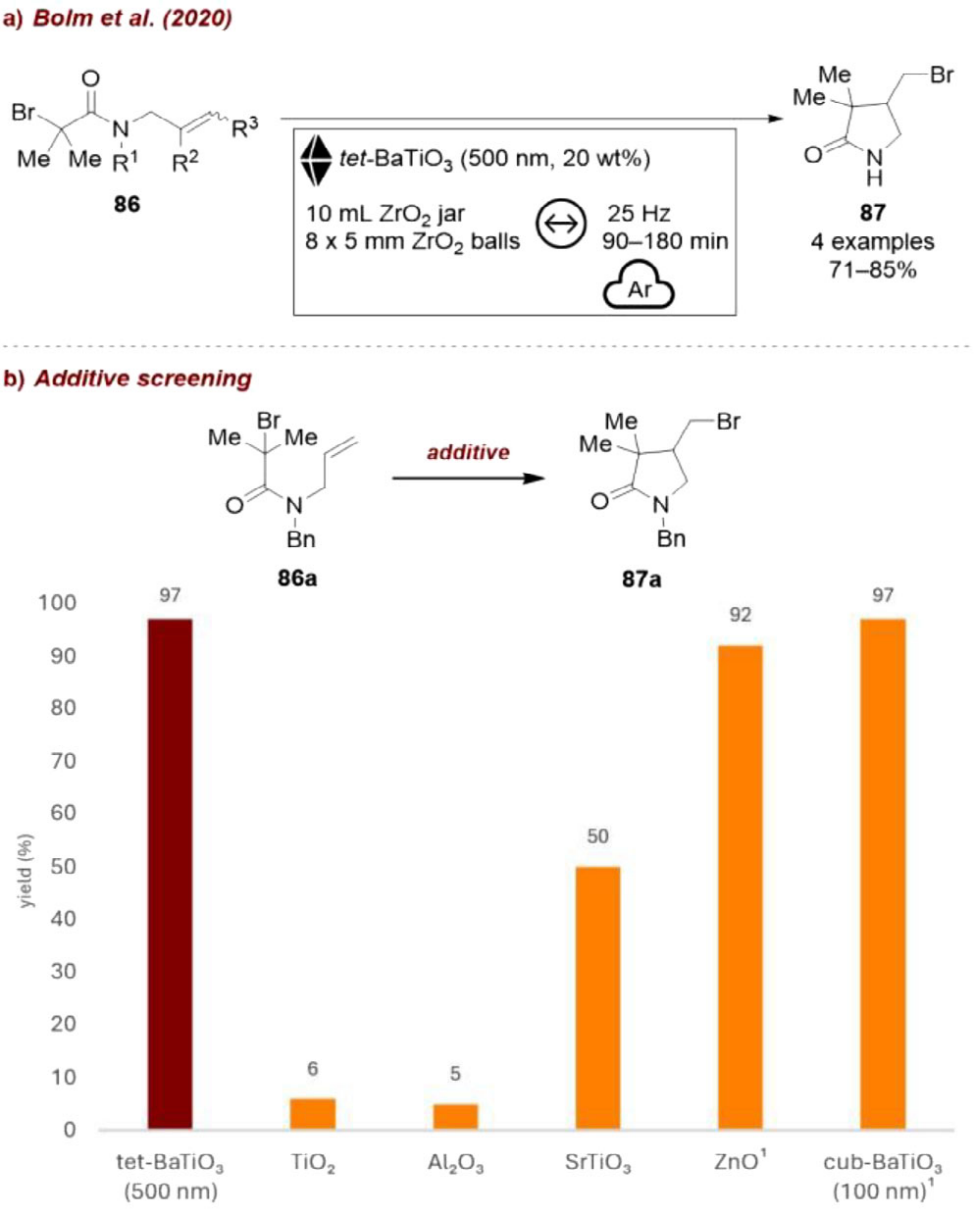
(a) Mechanochemical BaTiO_3_‐mediated atom transfer cyclization (ATRC) reaction. (b) Additive screening. Reaction conditions: **86a** (0.34 mmol), Cu(OTf)_2_ (5.0 mol%), TMPA (4.5 mol%), *additive* (20 wt%), 10 mL ZrO_2_ jar, 8×5 mm ZrO_2_ balls, mixer mill: 25 Hz, 90 min, under Ar atmosphere. Yield determined by ^1^H NMR as ratio of product vs. starting material. ^1^ 40 wt% added.

Soon after, Bolm et al. [124] reported a BaTiO_3_‐mediated atom transfer cyclization (ATRC) reaction (Figure 39a). They tested tetragonal and cubic BaTiO_3_, TiO_2_, Al_2_O_3_, SrTiO_3_, and ZnO (Figure 39b). Among the tested additives, BaTiO_3_ and ZnO performed best, giving above 90% ^1^H NMR ratio vs. starting material. Interestingly, cubic BaTiO_3_, which is intrinsically nonpiezoelectric due to its centrosymmetric crystal structure, could be activated with ball milling in a mixer mill and promoted the ATRC in an excellent yield of 97% when added in 40 wt%. As described earlier, even though nanosized BaTiO_3_ appears cubic and centrosymmetric at room temperature, local displacements of Ti^4+^ of up to ∼15 Å have been observed, which enable cubic nanosized BaTiO_3_ to exhibit piezoelectricity. This work serves as the first example of nanoscale cubic BaTiO_3_ becoming polarized and thus promoting an organic transformation under mechanical agitation. The authors chose tetragonal BaTiO_3_ as the piezoelectric additive, since only 20 wt% of the material was sufficient to deliver comparable results.

**FIGURE 40.**
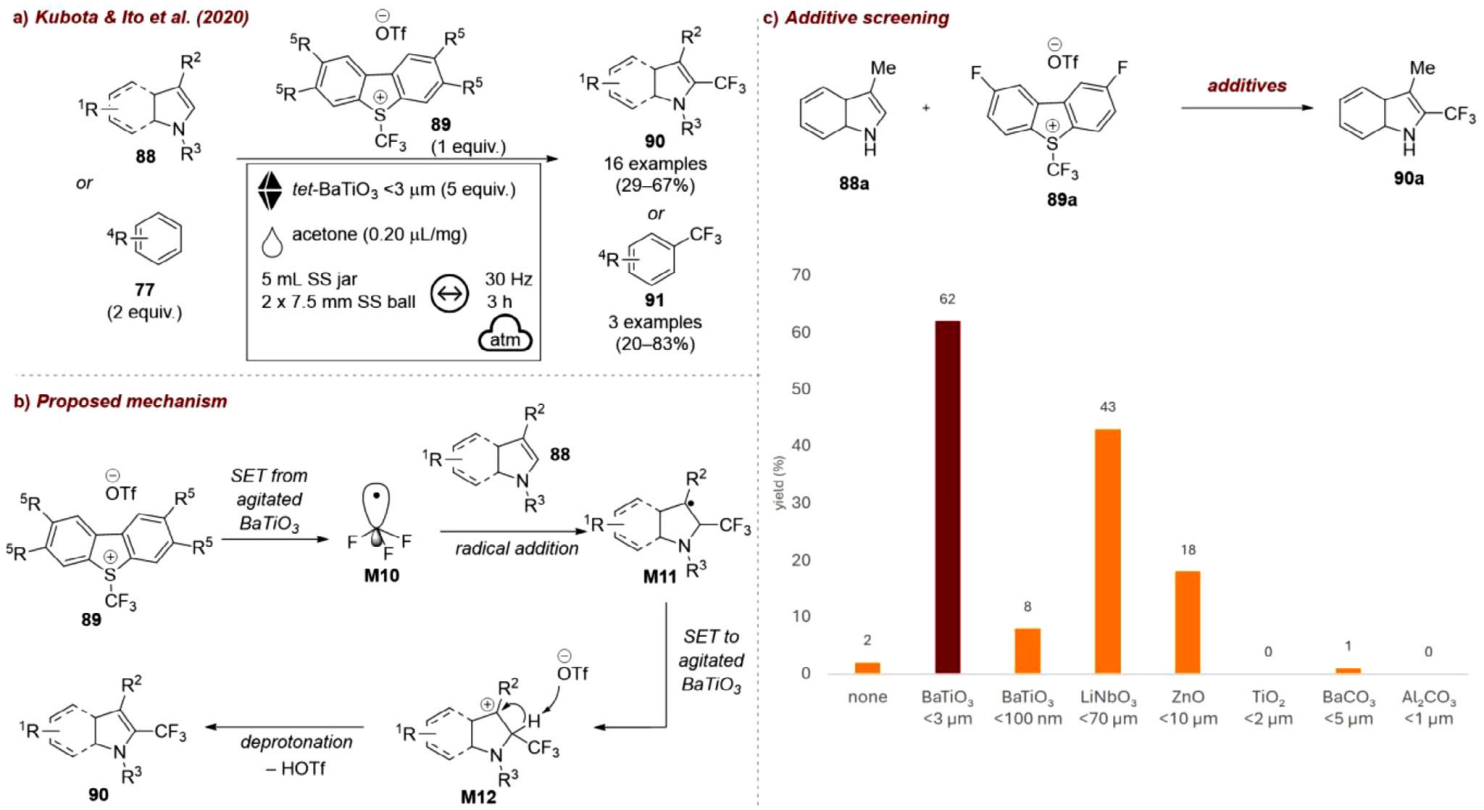
a)Mechanochemical radical C–H trifluoromethylation of *N*‐heterocycles and electron‐rich arenes. (b) Proposed mechanism. (c) Additive screening. Reaction conditions: **88a** (0.6 mmol), **89** (0.5 equiv.), *additive* (2.5 equiv.), acetone (0.20 µL/mg), 1.5 mL SS jar, 5 mm SS ball, mixer mill: 30 Hz, 1.5 h.

In 2020, Kubota and Ito [125] reported the use of BaTiO_3_ as a piezoelectric mediator for the radical C–H trifluoromethylation of *N*‐heterocycles, electron‐rich arenes, and tryptophan‐containing peptides in a ball mill (Figure 40a). In their screening studies, in the presence of acetone as LAG agent, they tested three piezoelectric materials: tetragonal BaTiO_3_, LiNbO_3_, and ZnO and three nonpiezoelectric materials: TiO_2_, BaCO_3_, Al_2_CO_3_ of different particle sizes (Figure 40c). They also tested cubic BaTiO_3_ (<100 nm). In the absence of any additive, trace amount of product was formed, while tetragonal BaTiO_3_ afforded a 62% NMR yield. In the presence of cubic BaTiO_3_ only 8% NMR yield was observed, and LiNbO_3_ and ZnO gave a 43% and 18% NMR yield, respectively. In the presence of nonpiezoelectric additives, no or only trace amounts of product were observed. The authors proposed a mechanism in which reduction of the Umemoto reagent **89** yields a CF_3_ radical that adds to the aromatic substrate, forming a CF_3_ radical **M10** (Figure 40b), which can add to aromatic **88**, furnishing trifluoromethylated intermediate **M11**. This species is then oxidized by a hole generated within the mechanically agitated BaTiO_3_ lattice (**M12**), and subsequent deprotonation furnishes the desired trifluoromethylated product **90**. Optimization studies further revealed that larger milling balls and jars improved product yields. When the reaction was performed under ultrasonic sonification, only trace amount of product **90** was observed. Together, these results strongly support the authors’ hypothesis that high‐impact ball milling is required to generate sufficient piezoelectric potential to drive the radical transformation.

**FIGURE 41:**
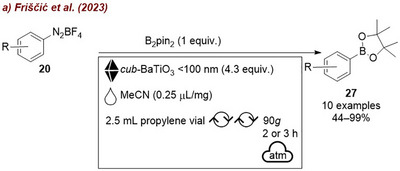
Mechanochemical activation of aryl diazonium salts for borylation reactions with *cub*‐BaTiO_3_.

Building on the work of Kubota and Ito [122], Friščić [126] reported the borylation reaction in the presence of cubic BaTiO_3_ NPs (<100 nm, Figure 41). Intrinsically nonpiezoelectric cubic BaTiO_3_ could be activated with RAM, in the absence of direct mechanical impact from milling media to promote the redox reaction. As in the ball‐milling method, the RAM method required MeCN as a liquid additive to achieve 92% NMR conversion. Notably, the RAM protocol also offers improved efficiency, providing the desired product in 2 h, compared with 3 h for the ball‐milling method [122] (Figure 38a). The recyclability of BaTiO_3_ was examined under shortened reaction times, and the authors observed a decrease in product yield from 68% in the first cycle to 11% in the fourth cycle, with mass losses of 5%–10% between cycles.

#### 1.9.2 Strontium Titanate (SrTiO_3_)

**FIGURE 42:**
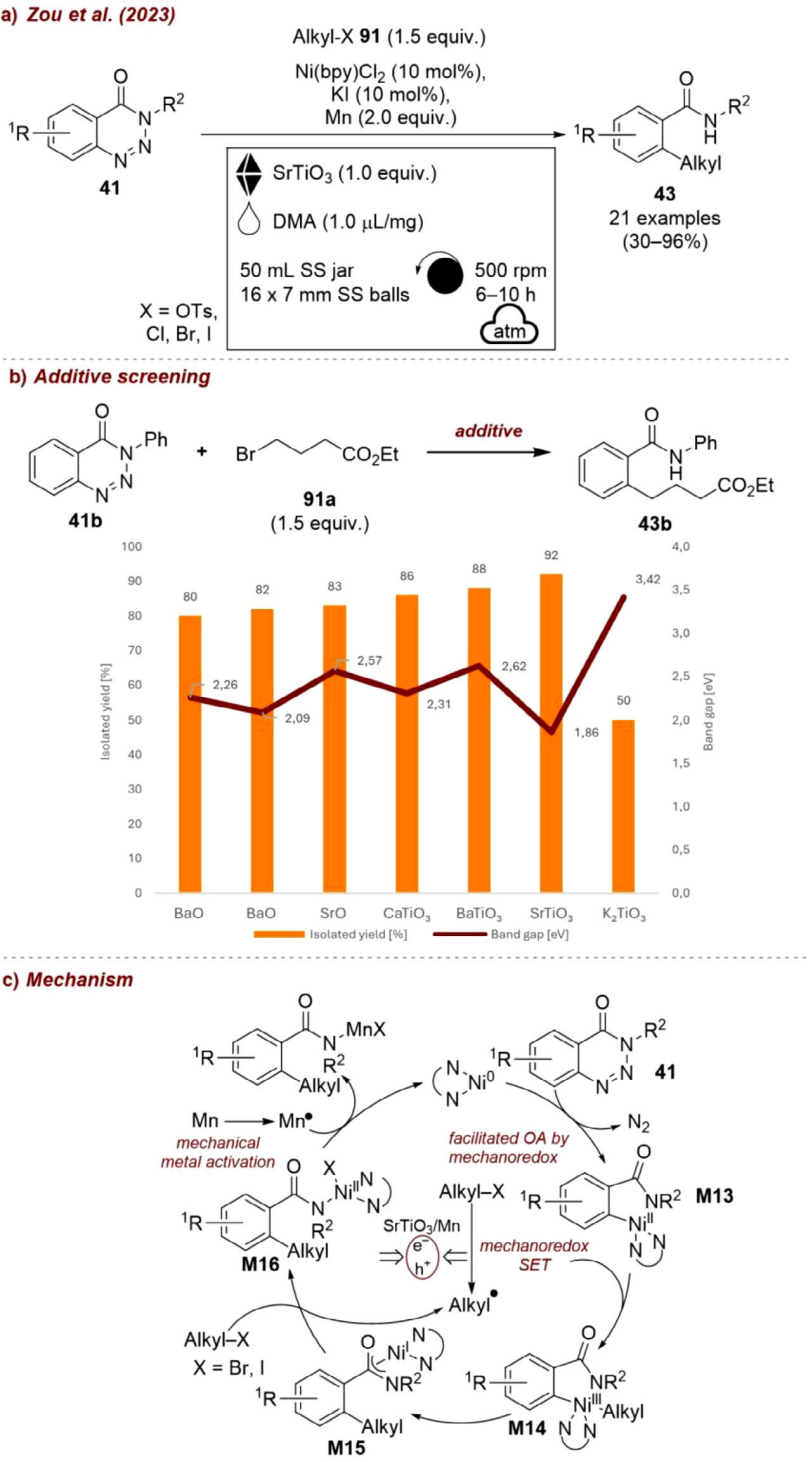
(a) Mechanoredox/nickel co‐catalyzed, manganese‐mediated cross‐electrophile coupling of benzotriazinones with alkyl(pseudo)halides. (b) Additive screening. Reaction conditions: **41b** (1 mmol), **91a** (1.5 equiv.), Ni(bpy)Cl_2_ (10 mol%), KI (10 mol%), Mn (2.0 equiv.), *additive* (1.5 equiv.), DMA (1.0 µL/mg), planetary mill: 500 rpm, 6 h. BaO from BaCO_3_, CaO from CaCO_3_, and SrO from SrCO_3_.

Tetragonal BaTiO_3_, most widely used form for organic mechanoredox reactions, is intrinsically polarized, which allows for lower forces for its activation. On the other hand, ambient temperature‐stable strontium titanate (SrTiO_3_) is cubic (centrosymmetric) and thus nonpolarized. For activation of these particles, high in‐plane strain is required to polarize the particles and further induce piezoelectricity. Zou et al. [127] reported an air‐tolerant mechanoredox/nickel co‐catalyzed, manganese‐mediated cross‐electrophile coupling of benzotriazinones **41** (Figure 42a) with alkyl (pseudo)halides **91**. This strategy utilized mechanical activation of Mn to eliminate chemical activators and mechanoredox co‐catalysis to overcome the low reactivity of alkyl (pseudo)halides under mild LAG conditions. To probe the role of piezoelectric materials as co‐catalysts, the authors tested several oxides. Carbonates (BaCO_3_, CaCO_3_, and SrCO_3_), which may undergo partial conversion into piezoelectric oxides under milling, gave access to **43b** in very good yields between 80 and 83% (Figure 42b). Remarkably, in the presence of typical piezoelectric additives such as BaTiO_3_, CaTiO_3_, and SrTiO_3_, the yield of **43b** increased to 86%−92%. In the control experiment with nonpiezoelectric K_2_TiO_3_ only 50% of **43b** was obtained, comparable to the experiment in the absence of any additive (54%). Notably, a general correlation was observed between product yields and band gaps in the additives: the higher the band gap, the lower the yield. Optimization studies revealed that 1.0 equiv. of SrTiO_3_ was sufficient, while lowering the additive loading to 0.5 equiv. resulted in a reduced yield of **43b**. Importantly, the piezoelectric additives can be reused, however a reduction in product yields was observed. These findings clearly demonstrate that mechanoredox co‐catalysis with piezoelectric materials significantly enhances the efficiency of the nickel‐catalyzed cross‐electrophile couplings under mild conditions. Reaction parameters such as rotational speed and jar filling degree also played a role. Consistently high yields of **43b** were achieved at 500–600 rpm, whereas reduction of the rotational speed results in incomplete conversion even after 10 h. The authors proposed a mechanism [128], in which the reactivity of benzotriazinones **41** increases due to ball‐milling‐induced mechanoredox catalysis, to facilitate the denitrogenative oxidative addition of Ni(0) to afford the five‐membered Ni(II) species **M13**, Figure 42c. Mechanical activation of SrTiO_3_/Mn allows for SET to the alkyl halide, forming an alkyl radical, which can add to **M13** after single‐electron oxidation by the generated hole in SrTiO_3_ to give **M14**. Following the intramolecular reductive elimination to form the aryl/alkyl bond in Ni(I) intermediate **M15**, a second single‐electron oxidation of Ni(I) to give Ni(II) species **M16**. Finally, mechanically activated Mn reduces complex **M16** to regenerate the catalytically active Ni(0) species. A control experiment with TEMPO confirmed a radical‐involved catalytic pathway.

#### 1.9.3 Piezoelectric MOFs

**FIGURE 43:**
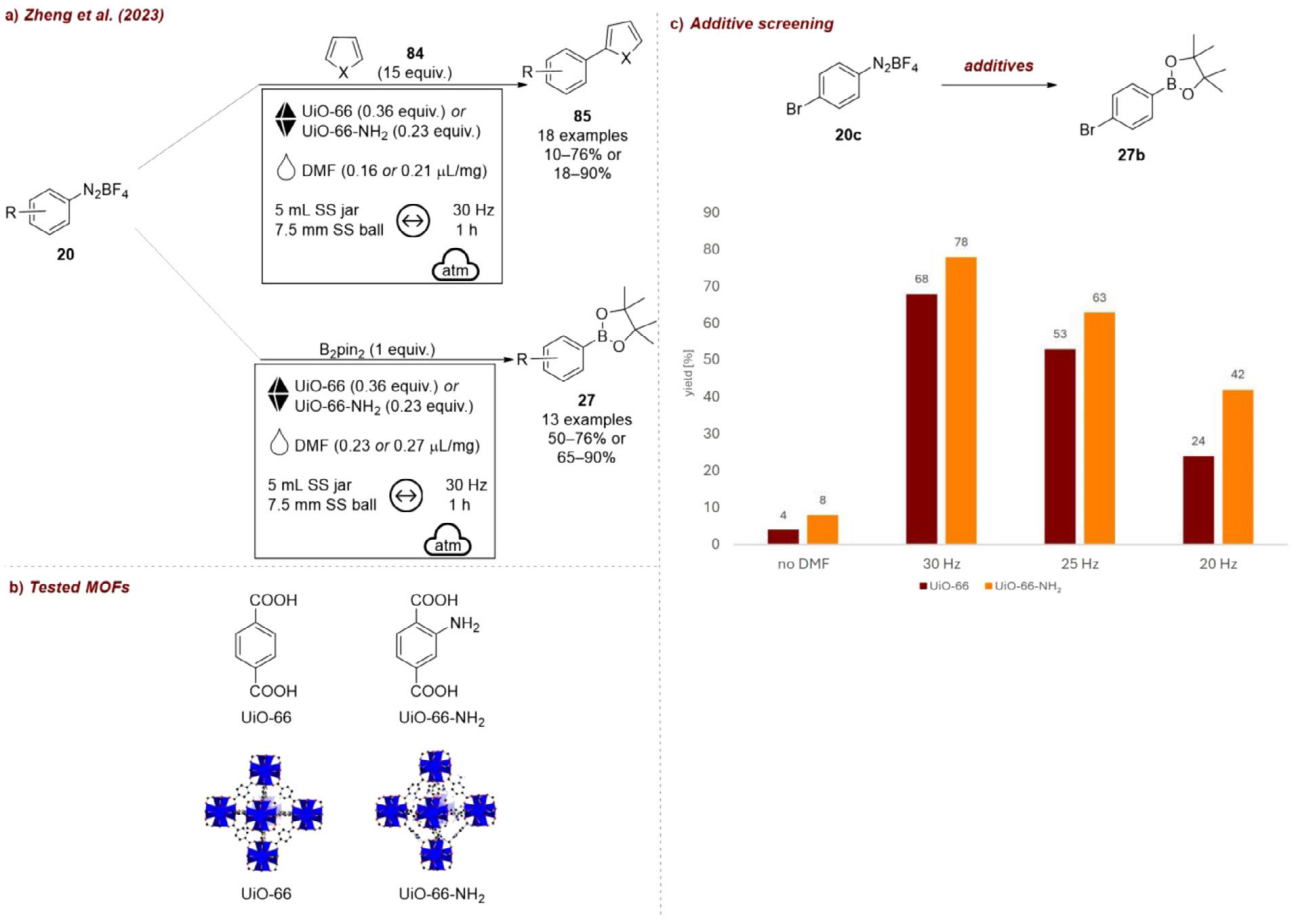
(a) Mechanochemical activation of aryl diazonium salts for heteroarene coupling and borylation reactions with piezoelectric MOFs. (b) Top: Chemical structure of ligands of MOFs UiO‐66 and UiO‐66‐NH_2_; Bottom: crystal structures. Crystal structures adapted from ref [127] with permission from American Chemical Society. (c) Additive screening. Reaction conditions: **20c** (0.3 mmol), B_2_pin_2_ (1 equiv.), UiO‐66 (0.36 equiv.) *or* UiO‐66‐NH_2_ (0.23 equiv.), DMF: 0.23 µL/mg *or* 0.27 µL/mg, 5 mL SS jar, 7.5 mm SS ball, mixer mill: *frequency*, 1 h. Yield determined ^1^H NMR.

The group of Zheng [129] reported the first application of piezoelectric MOFs to promote organic redox reactions under ball‐milling. The authors studied the well‐established reaction of diazonium salts **20** with heteroarenes **84** and with B_2_pin_2_ (Figure 43a) in the presence of two structurally stable, inexpensive, and easy‐to‐handle Zr‐based MOFs—UiO‐66 (Figure 43b) and UiO‐66‐NH_2_. Both materials exhibited similar pore‐size distributions and comparable particle morphologies, but their properties differed significantly: UiO‐66 displayed a modest piezoelectric response of (d_33_ = 16 pm/V), whereas UiO‐66‐NH_2_ showed a much stronger effect (d_33_ = 62 pm/V). The superior catalytic performance was attributed to the longer Zr‐O bonds, which generate higher polarity than in UiO‐66. Under neat conditions, both UiO‐66 and UiO‐66‐NH_2_ gave low yields of the borylated product (4% and 8%, respectively, Figure 43c). In the presence of DMF as a LAG additive, yields improved dramatically, reaching 67% for UiO‐66 and 78% for UiO‐66‐NH_2_. Milling frequency was also found to be critical: reducing the frequency from 30 Hz to 25 and 20 Hz resulted in progressively diminished yields. Recyclability studies showed a pronounced loss in crystallinity for UiO‐66‐NH_2_, and its piezoelectric response decreased dramatically from 62 pm/V to 4 pm/V indicating the close relationship between the piezoelectric activity and crystal size and shape. In contrast to traditional ceramic piezoelectrics such as BaTiO_3_ and SrTiO_3_, the MOFs and could be easily regenerated by treatment with concentrated HCl at 120 °C, restoring their octahedral morphology and thus catalytic activity. The substrate scope study revealed that UiO‐66‐NH_2_ mediated borylation reactions proceeds more efficiently and with higher yields than the previously reported BaTiO_3_ methods, underscoring the promise of piezoelectric MOFs as tunable alternatives to conventional inorganic piezocatalysts.

### 1.10 Mechanoluminescent Materials

Mechanoluminescence (ML) is the emission of light from a solid material as a response of mechanical stimuli such as stress, strain, friction, or deformation, without thermal excitation [130, 131]. ML is an umbrella term for several distinct modes of mechanically triggered luminescence and despite the considerable attention these materials are receiving, the exact mechanisms of light emission are still not fully understood. This review focuses exclusively on elastic‐mechanoluminescent materials (EMLs), the only subtype of ML that has been successfully applied in mechanochemical organic synthesis. EML, also known as piezoluminescence, arises from elastic deformation of noncentrosymmetric (piezoelectric) crystals, typically lanthanide‐doped salts such as aluminates, silicates, gallates, or titanates. Compared to plastic or fracture‐induced ML, EML requires lower stress and is fully reversible. Two main mechanisms have been proposed for EML, and those interested are referred to specialized review articles [132].

**FIGURE 44:**
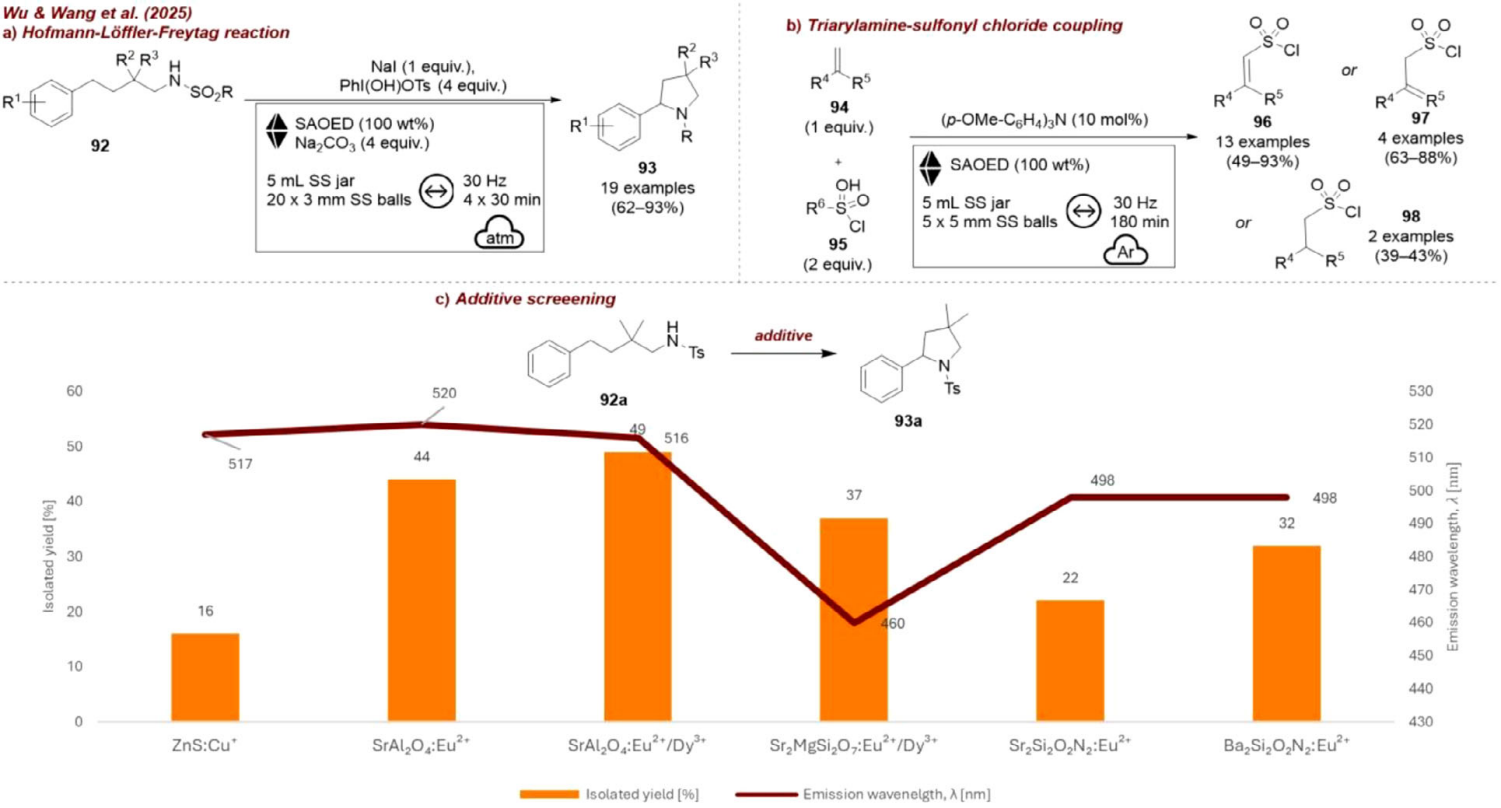
(a) Photomechanochemical Hofmann‐Löffler‐Freytag reaction and (b) Triarylamine–sulfonyl chloride coupling. (c) Additive screening. Reaction conditions: **92a** (1 equiv.), NaI (1 equiv.), PhI(OCOCF_3_)_2_ (4 equiv.), 5 mL SS jar, 20×3 mm SS balls, mixer mill: 30 Hz, 90 min.

Very recently, Wu and Wang [133] achieved the first photomechanochemical transformation, which utilizes an EML material: europium(II)‐ and dysprosium(III)‐doped strontium aluminate (SrAl_2_O_4_:Eu^2+/^Dy^3+^, SAOED) as internal light source for the mechanochemical Hofmann–Löffler–Freytag reaction (HLF, Figure 44a). Their method could even be expanded to excite electron donor‐acceptor (EDA) complexes, using Li and co‐worker's [134] triarylamine–sulfonyl chloride system (Figure 44b). The group tested 6 ML powders with suitable emission wavelengths (Figure 44c): ZnS:Cu^+^ (λ_ML_ = 517 nm), SrAl_2_O_4_:Eu^2+^ (λ_ML_  =  520 nm), SrAl_2_O_4_:Eu^2+^/Dy^3+^ (λ_ML_  =  516 nm), Sr_2_MgSi_2_O_7_:Eu^2+^/Dy^3+^ (λ_ML_  =  460 nm), SrSi_2_O_2_N_2_:Eu^2+^ (λ_ML_  =  498 nm) and BaSi_2_O_2_N_2_:Eu^2+^ (λ_ML_  =  498 nm). The efficiency of the additives was dependent on their ability to be activated by mechanical force, as well as their emission wavelength. Product yields ranged from 16% to 49%, with SAOED delivering the best results. Further experiments showed that employing Koser's reagent (PhI(OH)OTs) as oxidant and 4 equiv. of Na_2_CO_3_ as bulking additive further improved the yield to 84%. According to the authors, Na_2_CO_3_ probably acts as a base, facilitating the deprotonation of the N–H bond in the final intramolecular S_N_2 step. The investigation of the influence of the milling parameters showed that multiple smaller balls outperformed a single larger ball. This effect was attributed to the low excitation threshold of SAOED, which allowed even smaller balls to induce luminescence due to more frequent impacts and better energy distribution. While larger balls generated more intense emission per collision, their lower impact frequency reduced the overall efficiency. Further, both shortened and extended milling times reduced yields, resulting in incomplete conversion or side reactions and product decomposition due to heat accumulation during prolonged milling. This issue was resolved by adjusting the milling process to four 30 min intervals with 5 min breaks, which reduced heat buildup and increased the yield to 90%. With this application, the technical limitations of photomechanochemistry such as inefficient irradiation by external light sources and poor chemical and mechanical resistance of transparent poly(methyl methacrylate) (PMMA) jars were overcome, as the ML powder can simply be added directly to the SS reaction vessel. However, the application of SAOED for photomechanochemical reactions still exhibits some drawbacks, namely high additive amounts and limited recyclability. While the additive could be recycled, a gradual reduction in yield was observed for the sulfonylation reaction; from 93% in the first cycle to 70% in the fourth cycle even after calcination at 700 °C to remove organic residues from SAOED.

## 2 Conclusion and Outlook

From a practical perspective, solid ionic and non‐ionic additives are particularly recommended when liquid reagents or intermediates are involved, thus serving as bulking agents. As demonstrated in the discussed examples, they alter the material rheology and can thus significantly enhance reaction efficiency. However, surpassing the mere role of bulking agents they have also demonstrated their ability to control reactivity and selectivity, which at times would be inaccessible through traditional approaches. More specialized additives, such as ILs, piezoelectric materials, and mechanoluminescent solids, are expected to open new mechanistic possibilities and expand the reactivity space of mechanochemistry even further.

The ability to tune reaction concentration and selectivity without relying on hazardous organic solvents offers an additional advantage of additive‐assisted mechanochemical synthesis. Nevertheless, while mechanochemistry minimizes or eliminates solvent use and therefore significantly reduces waste generation, the deployment of additives in stoichiometric or even superstoichiometric quantities introduces a new sustainability challenge. To ensure that additive‐assisted grinding remains viable for industrial‐scale applications, the recyclability and reusability of these materials must be a major focus in developing new reactions and methods.

Overall, additives demonstrate a multifaceted impact beyond traditional grinding auxiliaries. Their physicochemical properties most of the time directly influence reaction outcomes, selectivity, and reproducibility. Despite their widespread use, understanding the actual mechanistic impact of the additives remains in its early stages and additive choice remains largely empirical. This underscores the need for systematic studies to clarify structure–function correlations and to enable more predictive additive selection. Optimizing additive loading was shown to be a crucial step for balancing efficient energy transfer with proper reagent interaction, achieving optimal results. At present, no standardized framework for additive choice exists and it remains unclear whether such a universal concept is even attainable. A deeper understanding of reaction mechanisms and how they diverge from solution‐phase chemistry, or even from mechanochemical reactions employing other additives, remains of central importance. As demonstrated throughout this review, additives consistently influence mechanochemical transformations and will undoubtedly continue to drive innovation in the field. Broader screening efforts are likely to reveal additional reactivity modes and will aid in the rational design of next‐generation mechanochemical processes.

To address this central drawback, major progress is expected from the development of direct time‐resolved in‐situ (TRIS) monitoring techniques, such as X‐ray powder diffraction (XRD), X‐ray absorption fine structure (XAFS), Raman spectroscopy, and nuclear magnetic resonance (NMR) spectroscopy. These TRIS techniques, developed only in the last decade, have already contributed substantially to our understanding of mechanochemical transformations [135, 136, 137]. In the context of the present review, it would be highly beneficial for researchers to focus not only on the phase composition, crystallite size, and temperature during milling, but also elucidating the role of additives in these processes.

Once more comprehensively understood and widely adopted, additive‐assisted grinding has the potential to transform not only laboratory‐scale synthesis but also industrial manufacturing, polymer recycling, and environmental remediation, ultimately contributing to more sustainable and efficient chemical technologies.

## Conflicts of Interest

The authors declare no conflicts of interest.
